# Benchmarking validity indices for evolutionary K-means clustering performance

**DOI:** 10.1038/s41598-025-08473-6

**Published:** 2025-07-01

**Authors:** Abiodun M. Ikotun, Faustin Habyarimana, Absalom E. Ezugwu

**Affiliations:** 1 School of Mathematics, Statistics and Computer Science, University of KwaZulu- Natal, KwaZulu-Natal, Pietermaritzburg Campus, Durban, South Africa; 2 Unit for Data Science and Computing, North-West University, 11 Hoffman Street, Potchefstroom, 2520 South Africa

**Keywords:** Clustering algorithms, Automatic clustering, Metaheuristic optimisation, K-means, Cluster validity indices, Evolutionary k-means, Evolution, Mathematics and computing

## Abstract

K-Means is a well-established clustering algorithm widely used in data analysis and various real-world applications. However, its requirement for a predefined number of clusters limits its effectiveness in automatic clustering tasks. To address this, metaheuristic optimisation algorithms have been integrated into K-Means, leading to the development of Evolutionary K-Means clustering approaches. These methods often rely on internal validity indices as fitness functions to automatically determine both the optimal number of clusters and the clustering configuration. However, the effectiveness of internal validity indices is often data-dependent, as most are tailored to specific data characteristics. Consequently, the choice of validity index can significantly influence clustering outcomes. This study evaluates the performance of fifteen internal validity indices within the Enhanced Firefly Algorithm-K-Means (FA-K-Means) framework, an evolutionary approach that integrates Firefly metaheuristics with the classical K-Means algorithm. The performance of each index is assessed across a diverse collection of real-life and synthetic datasets with varying structures. The results reveal that the Calinski-Harabasz (CH) and Silhouette indices consistently outperform others, offering more reliable clustering performance. These findings provide practical guidance for selecting appropriate fitness functions in Evolutionary K-Means algorithms for automatic clustering tasks.

**Supplementary Information:**

The online version contains supplementary material available at 10.1038/s41598-025-08473-6.

## Introduction

Cluster analysis is one of the most important and primitive activities in exploratory data analysis. The primary goal of data clustering is to uncover hidden similarities among objects within an unlabelled dataset and group them based on these latent structures. As a fundamental technique in unsupervised machine learning, clustering is vital in extracting meaningful patterns and intrinsic information from unlabelled data, making it essential for various data analysis tasks. Its applications span diverse fields, including web data analysis, text mining, data exploration, and bioinformatics, particularly in the context of big data. However, a significant limitation of many traditional clustering algorithms is the requirement to predefine the number of intrinsic groups in the dataset, which is often impractical when dealing with large, complex, or unknown datasets. Automatic clustering algorithms have been developed to address this limitation. These algorithms are designed to automatically determine the optimal number of clusters in unlabelled datasets and partition the data into the identified groups based on intrinsic characteristics of the dataset, thereby overcoming the challenges associated with manual cluster number specification.

Automatic clustering algorithms detect hidden clusters in unlabeled datasets using internal cluster validity indices. The algorithms employ the internal cluster validity index as an objective function that needs to be optimised in the search for the optimal number and partitioning of the unlabelled datasets. Cluster validation identifies optimal partitioning of unlabelled datasets that aligns with the natural divisions inherent in the dataset without any reliance on any class information while performing the clustering task^1^. In cluster validation, the goodness of the resulting clusters obtained from the clustering is measured using the underlying dataset structure without any prior information about the dataset. It also evaluates the performance of clustering algorithms employed for grouping the data.

The K-means clustering algorithm is widely known and used for its easy usage, effectiveness and simplicity^2^. However, its functionality is limited when handling automatic clustering challenges. K-means requires that the number of clusters is specified as part of initial parameters and the manual specification of the corresponding initial cluster centres. The basic characteristics associated with big data, such as data sample complexities in terms of high dimensionality, presence of outliers and noise, and irregular sample distribution of data points in the datasets, further affect the clustering performance of the K-means algorithm. As part of ongoing research to resolve this k-means dilemma, the K-means algorithm is being integrated with many metaheuristic optimisation algorithms^2–5^ to enhance its clustering performance and expand its capability in handling automatic clustering tasks.

Integrating the K-means algorithm with metaheuristic-based optimisation algorithms resolves several challenges of the traditional K-means algorithm. The powerful investigative skill based on the ability of the metaheuristic optimisation algorithms to explore and exploit the search space assists in the selection of optimum initial cluster centroids, thereby resolving the problem of local optimal entrapment^3^ of the K-means algorithm. Evolutionary K-means clustering algorithms combined with various metaheuristic algorithms have been experimentally proven to be highly effective and reported in the literature. Different metaheuristic algorithms, such as the hybrid Gray wolf optimiser^4^, Hybrid Symbiotic Optimization Search Algorithm, hybrid fruit fly optimisation algorithm^5^, Hybrid Firefly algorithm, and hybrid multi-verse optimiser^6^, among many others, have been paired with K-means for solving clustering related tasks. A review article^7^ discussed nature-inspired metaheuristic algorithms integrated with K-Means for automatic clustering. Recent hybridisation of metaheuristic algorithms with K-means for automatic clustering includes^8–10^.

Enhanced FA-K-means is one of the most recent hybridised clustering algorithms that integrated the K-means algorithm with the firefly metaheuristic algorithm (FA)^10^. The Enhanced FA K-means combined an improved version of the firefly algorithm with an enhanced K-means algorithm. As a population-based nature-inspired meta-heuristic optimisation algorithm, the firefly algorithm^11^ mimics the behaviour of fireflies to solve complex optimisation problems. Based on its unique automatic subdivision capability, it can solve multimodal optimisation problems, such as the clustering problem^12^. In the traditional K-means, the number of distance calculations usually increases as the number of clusters increases. The computational complexity of the K means algorithm, dependent on the dataset’s size and dimensionality, is thus affected. The enhanced FA-k-means addressed this distance calculation challenge of the standard K-means. In the Enhanced FA-K-means algorithm, the firefly algorithm automatically determines the optimal number of clusters and generates the corresponding initial cluster centroids. These parameters are the initial inputs for the modified K-means algorithm, which incorporates the central limit theorem for efficient distance computation. The Enhanced FA-K-means algorithm has been statistically validated to outperform other hybridised K-means variants, demonstrating superior clustering accuracy while significantly reducing computational time^10^.

The performance of clustering algorithms is affected significantly by several factors, including cluster overlap, high dimensionality, number of clusters and imbalance in cluster sizes^1^. Moreover, most validity indices are designed to address certain data types, making their performance data dependent^13^. As a result, selecting a specific internal validity index may critically affect the quality of the resultant clusters. Selecting and applying the most suitable CVs for a specific clustering algorithm, particularly when working with datasets of varying characteristics, can significantly enhance the quality of the clustering results produced by that algorithm. This study evaluates and compares the performance of fifteen cluster validity indices on real-life and synthetic datasets with diverse characteristics using the Enhanced Firefly-K-means clustering algorithm framework.

In this paper, Fifteen different internal cluster validity indices, including Davis-Bouldin Index (DBI)^14^, Calinski-Harabasz Index (CHI)^15^, Compact Separated Index (CSI)^16^, Dunn Index (DI)^17^, General Dunn Index (GDI)^18^, S_Dbw Index^19^, Silhouette Index (SI)^20^, Xie-Beni index (XBI)^21^, COP Index^22^, Sym-Index^23^, SV Index^24^, Pakhira-Bandopadhyah Maulik Index (PBM)^25^, Score Function Index (SF)^26^, SymDB Index^22^, Point Symmetry Index (PSI)^27^ were used in the experimental study. Also, the study aims to identify which CVI performs most effectively across different categories of datasets on an evolutionary-K-means clustering framework for automatic clustering. This study will provide valuable insights that will assist researchers and practitioners in selecting appropriate fitness functions for evolutionary K-means, especially the base algorithms, when deployed for automatic clustering tasks in real-life applications. This analysis used twelve real-life and synthetic datasets with diverse characteristics, such as non-linearly separable clusters, arbitrarily overlapping shaped clusters and those with complex path clusters. Both the real-life and synthetic datasets are selected as a representation of datasets that may be prevalent in real-life applications.

The remainder of this paper is structured as follows: the related works are presented in Sect. 2. Section 3 briefly discussed the different CVIs used in the experimental study and the Enhanced FA-K-means algorithm. Section 4 reports the experimental setup and simulation, as well as the report evaluation and discussion. Finally, Sect. 5 presents the conclusion and the suggestion for future work.

## Related work

This section considers the various CVIs mostly used in metaheuristic optimisation algorithms as objective functions and specifically in Evolutionary K-means algorithms for automatic clustering existing in literature^28^. The different existing comparison studies on these CVIs are also reported. The metaheuristic optimisation algorithms for automatic clustering use CVIs to identify the collection of clusters inherent in a dataset without any reliance on any prior knowledge relating to the dataset. It uses the underlying architecture of the dataset to evaluate the cluster’s goodness. It is used primarily in automatic clustering to determine the optimal number of clusters in a dataset and evaluate the performance of the clustering algorithm on any specific dataset.

Several CVIs have been employed by researchers as fitness functions in metaheuristic optimisation algorithms for automatic clustering. In^29^, the CS index was used as the CVI for automatically clustering real-life datasets based on a quantum-inspired differential evolution algorithm. A novel cluster validity index that is tailored to the features and objectives of multiple model control (MMC) schemes was proposed in^30^ for a genetic-based automatic clustering approach for their MMC. The authors combined the DBI, CSI and SI in^31^ as a framework for merging several validity indices into a single measure for a collaborative decision support system for multi-criteria automatic clustering. The authors combined the three CVIs as a multiplication of the normalised functions of the CVIs to the power of their respective weight of importance to serve as their objective function model.

The CSI and DBI indices were used in^32^ for the dynamic Social Particle Swarm Optimization for automatic clustering. The authors adopted the xie-Beni index, correlation-based cluster validity index and object-based cluster validity index in^33^ for their multi-level quantum-inspired metaheuristic for automatic clustering of hyperspectral images. The DBI and CSI were used by^34^ in the swarm-based automatic clustering in their proposed nature-inspired Emperor Penguins Colony algorithm. Several evolutionary K-means algorithms that use different internal validity indices have been proposed in the literature. A hybrid genetic-fuzzy ant colony optimisation algorithm for automatic k-means clustering (HGA-FACO)^35^ used the Silhouette Coefficient (SC), Partition Coefficient (PC), Davies Bouldin (DB) and Sum of Squared Errors (SSE). The PBM validity index was used in^36^ for automatic clustering based on the dynamic parameter’s harmony search optimisation algorithm(AC-DPHS), which combines the K-means and dynamic parameters harmony search for data clustering.

The authors in^37^ used the DBI for their quantum-inspired genetic algorithm for k-means clustering. DB and XB indexes were used in^38,39^. In^40^, the proposed niche GA hybridised with K-means used PBM, SSE, DB and XB. The CSI index was used by Deep^41^ for the automatic initialisation in K-means combined with the artificial hummingbird algorithm. In the work reported by^42^, the Silhouette coefficient index was used to check the different clustering and choose the best cluster number for their automatic clustering algorithm that hybridised K-means with harmony search and artificial bee colony optimisers. In^43^, nineteen internal cluster validity indices and three external cluster validity indices were used to evaluate the clustering performance of the proposed collaborative annealing power K-means + + clustering.

There is existing literature that has reported hybridisation with Firefly algorithm for data clustering. The authors in^44^ used CS and DB as fitness functions for their FAPSO algorithm. The authors used the CS and DB index in^45^ for five hybrid metaheuristic algorithms involving firefly optimisation algorithms for a comparative analysis of the performance of the different firefly-based hybrid algorithms. In some of the above-mentioned automatic clustering tasks involving two or more CVIs, the intent is to report the clustering performance of the underlying clustering algorithm and not specifically compare the performances of the CVIs.

There are specific research studies that focus on comparing the performance of different CVIs using different metaheuristic-based clustering algorithms^1,13,46,47^. Some comparative analyses used the CVIs as external cluster validation where the final clustering results of each clustering task with different initial clustering parameters are presented as input into the CVIs to determine the clustering quality to identify the best. For comparisons involving the clustering algorithm that integrated CVIs as a fitness function, in most cases, comparisons are conducted to establish the superior performance of a new CVI or an enhanced version of an existing one.

There are existing comparison works that involve the analysis of the performance of CVIs as fitness functions in metaheuristic optimisation algorithms for automatic clustering. The authors in^48^ considered 68 CVIs in their comparison. The cluster results generated using the K-means clustering algorithm on 21 simulated and real-life datasets were evaluated and compared. The comparison used the same partition for each dataset. This differs from this study because it is not an automatic clustering task. After all, the classical k-means were employed to partition the datasets. Moreover, the CVIs are used as an external validity index to validate standard partitions generated via the K-means clustering.

The performance of two internal cluster validation criteria- the BetaCV and Dunn index- were evaluated and compared in^49^. The CVIs were used as cost functions to guide the agents of the swarm optimisers -PBO-BCV and PSO-Dunn- in searching for a suitable cost function for a successful clustering outcome. In^50^, the effectiveness of 22 different cluster validity was evaluated to identify the scenario in which the CVIs perform well and to identify their limitations. An evolutionary clustering algorithm, ACDE, was used as the clustering algorithm platform for the experiment. Both synthetic and real-life datasets were used to present varied dataset characteristic scenarios for the evaluation.

In^51^, 52 CVIs were compared to establish the proof that the CVIs are promoting clustering that appropriately matches expert knowledge. In this case, the CVIs are treated as fitness functions to find the optimal partition among a given cardinality’s possible partitions. This is different from this study because it is not about automatic clustering. However, it presents a study that confirms the authenticity of using CVIs as a fitness function to validate optimal clustering output. Closely related to our work is the comparison study of^13^, which evaluates and compares eight clustering validity indices on swarm intelligence-based clustering framework using differential-evolution particle swarm optimisation as the clustering algorithm.

This study evaluates and compares the performances of fifteen CVIs using the framework of an evolutionary K-means algorithm on twelve datasets with varying characteristics to identify appropriate validity indices in evolutionary K-Means clustering algorithms to provide insight into the selection of suitable internal validity index for similar automatic clustering algorithm framework when handling datasets with corresponding varying characteristics. This research distinguishes itself from prior studies in the following key aspects:

i. Enhanced FA-K-means Algorithm: Unlike previous works that primarily evaluate CVIs with standard clustering algorithms, our study introduces an enhanced Firefly Algorithm (FA)-K-means hybrid, which optimizes both convergence speed and cluster quality. This algorithmic innovation provides a more robust and efficient framework for CVI evaluation.
ii. Comprehensive CVI Coverage: While prior studies (e.g^1,14,45,46^)., focused on a subset of CVIs, our work systematically evaluates 15 internal CVIs—spanning classical (e.g., Silhouette, Davies-Bouldin) and recent advances (e.g., Score Function, S_Dbw). This breadth offers a more holistic comparison of validity indices under diverse conditions.
iii. Diverse and Challenging Datasets: Our benchmarking employs datasets with varying characteristics (e.g., high dimensionality, imbalanced clusters, and noise), including synthetic and real-world data (e.g., from UCI and OpenML). This ensures the evaluation reflects practical scenarios not fully addressed in earlier works.
iv. Unique Evaluation Criteria: Beyond traditional metrics like accuracy, we assess CVIs based on:
  Stability across multiple runs,
  Sensitivity to initialization and noise,
  Computational efficiency in large-scale settings,
  Robustness to cluster overlap and dimensionality. So, these criteria provide deeper insights into CVI performance for evolutionary clustering.
v. Explicit focus on Evolutionary Dynamics: While references^1,14,45^, and^46^ examined static clustering, our work explicitly evaluates how CVIs perform in iterative, evolving cluster environments, a gap in prior benchmarking studies.

## Methodology

This section describes the various internal cluster validity indices used in this study and the enhanced FAK-Means clustering algorithm for experimentation.

### Cluster validity indices

Cluster validity indices evaluate the quality of clustering solutions. Automatic clustering is also used to determine the optimum cluster numbers and the natural distribution of the groups (clusters) present in the dataset without prior knowledge of the dataset characteristics. A clustering solution is said to be of optimum quality if the clusters are well separated such that the members within a cluster are similar and distinctively different from the members of other clusters.

The internal CVIs measure the cluster’s compactness and cluster separation. Cluster compactness measures the homogeneity (tightness, cohesion, connectedness) of the cluster, while separation measures the heterogeneity of the different clusters. Cluster compactness is based on the intra-cluster distances between the objects within the same cluster, and the cluster separation is evaluated by measuring the inter-cluster distances between the dataset’s clusters.

Several internal CVIs have been reported in the literature. Still, this paper has considered a few to evaluate their performances on different datasets with diverse characteristics within the evolutionary K-Means algorithm’s framework for automatic clustering problems. The fifteen cluster validity indices used in the study are briefly discussed in this section. The information about the various CVIs used in the experimentation is discussed below.

Davis-Bouldin Index: The DB index finds the average ratio of the intra-cluster distance to the inter-cluster distance for all the possible pairing of the identified clusters. The DBI is defined using Eq. (4).

4 $$\:DBIndex=\:\frac{1}{c}\sum\:_{i}^{c}\underset{i\ne\:j}{\text{max}}\left\{\frac{{A}_{i}+{A}_{j}}{d(\stackrel{-}{{c}_{i}},\stackrel{-}{{c}_{j}})}\right\}$$

where $\:{A}_{i}$ is the mean distance of all the elements of $\:{c}_{i}$ and it’s center $\:\stackrel{-}{{c}_{i}}$.

5 $$\:{A}_{i}=\frac{1}{{m}_{i}}\sum\:_{j=1}^{{m}_{i}}d({c}_{j},\:\stackrel{-}{{c}_{i}})$$

Calinski-Harabasz Index: The CH index finds the ratio of the cluster’s connectedness to the cluster’s separateness. However, while the cluster’s connection is estimated based on the distance between the data points within the corresponding cluster’s centroid, the cluster’s separateness is estimated based on the distance of each cluster’s centre from the global centre. The CH index is defined using Eq. (5).

6 $$\:CHIndex=\frac{NF-NC}{NC-1}\:\frac{{\sum\:}_{a=1}^{NC}d\left({cc}_{a},\:GC\right)}{{\sum\:}_{a=1}^{C}\sum\:_{{x}_{b}\in\:{C}_{a}}d({x}_{b},{cc}_{a})}$$

7 $$\:{cc}_{a}=\:\frac{1}{{ND}_{a}}\sum\:_{{y}_{b}\in\:{C}_{a}}{y}_{b},\:\:\:\:a=1,\dots\:.,\:\:\:NC$$

8 $$\:GC=\:\frac{1}{ND}\sum\:_{b=1}^{ND}{y}_{b}$$

Compact Separated Index: The CS index^52^ finds the ratio of the sum of the intra-cluster distance to the sum of the inter-cluster distance. It seeks clusters with the minimum intra-cluster distance and maximum inter-cluster distance. The CS index is defined using Eq. (9).

9 $$\:CSindex=\:\frac{{\sum\:}_{i=1}^{g}\left[\frac{1}{\left|{B}_{i}\right|}{\sum\:}_{{u}_{j\in\:{B}_{i}}}\underset{{u}_{k}\in\:{B}_{i}}{\text{max}}\left\{d({u}_{j},{u}_{k})\right\}\right]}{{\sum\:}_{i=1}^{g}\left[\:{min}_{j\in\:g,j\ne\:i}\:\:\left\{d\left({v}_{i},{v}_{j}\right)\right\}\right]}$$

Dunn Index: In the Dunn index^52,53^, the minimal ratio of the distance between the closest objects of different clusters to the distance between the most separated objects within the same cluster (that is, the largest cluster diameter) is used in identifying well-separated clusters in a dataset. The Dunn index is defined using Eq. (10).

10 $$\:Dunnindex=\frac{\:{min}_{1\le\:i\le\:c}\left\{\underset{1\le\:j\le\:c;j\ne\:i}{\text{min}}\left\{\phi\:({C}_{i},{C}_{j})\right\}\right\}\:}{\underset{1\le\:k\le\:c}{\text{max}}\left(\omega\:\left({C}_{k}\right)\right)}$$

11 $$\:\phi\:\left({C}_{i},\:{C}_{j}\right)=\text{m}\text{i}\text{n}\left\{d\left({x}_{i},{x}_{j}\right)\::\:{x}_{i}\in\:{C}_{i},\:{\:x}_{j}\:\in\:{C}_{j}\right\}$$

12 $$\:\omega\:\left({C}_{k}\right)=max\left\{d\left({x}_{i},{x}_{j}\right)\::\:{x}_{i},{x}_{j}\in\:{C}_{k}\right\}$$

Generalised Dunn Index: The generalised Dunn index differs from the classical Dunn index in terms of the choice of the set distance used in the numerator and denominator of the index function. The Euclidean distance is used for the two functions in the classical Dunn index. $\:{\phi\:}_{a}$ and $\:{\omega\:}_{b}$. Other distance functions applicable in the GDunn index include the complete and average linkage functions^54^.

13 $$\:GDindex=\frac{\:{min}_{1\le\:i\le\:c}\left\{\underset{1\le\:j\le\:c;j\ne\:i}{\text{min}}\left\{{\phi\:}_{a}({C}_{i},{C}_{j})\right\}\right\}\:}{\underset{1\le\:k\le\:c}{\text{max}}\left({\omega\:}_{b}\:\left({C}_{k}\right)\right)}$$

S_Dbw Index: The S_Dbw index assesses the validity of clustering results by exploiting the inherent features of clusters. S_Dbw index considers cluster density alongside compactness and separation, which are the intra-cluster variance and the inter-cluster density. The S_Dbw is defined using Eq. (14).

14 $$\:{S}_{Dbwindex}=\:ClustScat\left({N}_{c}\right)+{Dens}_{btw}Clust\left({N}_{c}\right)$$

where:

15 $$\:{Dens}_{btw}Clust\left({N}_{c}\right)=\:\frac{1}{{N}_{c}.({N}_{c}-1)}\sum\:_{i=1}^{{N}_{n}}\left(\sum\:_{\begin{array}{c}j=1\\\:i\ne\:j\end{array}}^{{N}_{c}}\frac{density\left({m}_{ij}\right)}{\text{m}\text{a}\text{x}\left\{density\left({cc}_{i}\right),density\left({cc}_{j}\right)\right\}}\right)$$

16 $$\:density\left({m}_{ij\:}\right)=\:\sum\:_{g=1}^{{N}_{ij}}f({x}_{g},\:{m}_{ij})$$

such that:

$\:{N}_{c}$ = Number of clusters

$\:{cc}_{i}$, $\:{cc}_{j}$ = Cluster centre for $\:{C}_{i\:}and\:{C}_{j}\:$

$\:{m}_{ij}$ = the midpoint of the line segment defined by $\:{cc}_{i},\:{cc}_{j}$

$\:{N}_{ij}\:$= Number of tuples belonging to clusters $\:{C}_{i\:}and\:{C}_{j}$ in the neighbourhood of $\:{m}_{ij\:}$

Silhouette Index: The Silhouette index finds the validity of a clustering result using the cohesion and separation of the formed clusters. The cluster cohesion is estimated based on the mean distance of each element to all other elements in the same cluster. In contrast, the cluster separation is calculated as the smallest mean distance between each component of a cluster and all aspects in any other cluster. The Silhouette index is defined using Eq. (17).

17 $$\:Silindex=\:\frac{1}{n}\sum\:_{i=1}^{n}Z\left({x}_{i}\right)$$

where

18 $$\:Z\left({x}_{i}\right)=\:\frac{r\left({x}_{i}\right)-\:q\left({x}_{i}\right)}{\text{max}\left\{q\left({x}_{i}\right),\:\:r\left({x}_{i}\right))\right\}}$$

Xie-Beni Index: In Xie-Beni index (XBI)^48,55^ the ratio of the cluster cohesion to the cluster separation is calculated to estimate the validity of clustering results. The global average square distance of objects within a cluster from their cluster centroid determines cluster cohesion. In contrast, the minimum squared distance between pairs of clusters is used for cluster separation. The XBI is defined using Eq. (19). A smaller value of the Xie-Beni index indicates better results.

19 $$\:XBindex=\:\frac{\sum\:_{a=1}^{k}\sum\:_{b=1}^{n}{m}_{ab}^{f}\:{{\parallel}{x}_{b}-{g}_{a}\:{\parallel}}^{2}\:}{n{min}_{ab}\:{{\parallel}{g}_{a}-\:{g}_{b}{\parallel}}^{2}}$$

where:

$\:k$ = the number of clusters

$\:n$ = the number of data points

$\:{m}_{ab}$ = membership value of object $\:b$ with group centre $\:a$

$$\:f$$ = fuzzifier

$\:{\parallel}{x}_{b}-{g}_{a}\:\parallel$ = Euclidean distance of data point ($\:{x}_{b})$ with the group centre ($\:{g}_{a})$

$\:\parallel{g}_{a}-{g}_{b}\:\parallel$ = group centres Euclidean distance

COP Index: The COP index finds the ratio of the intra-cluster distance to the inter-cluster distance in estimating the validity of a clustering result. While the intra-cluster distance is measured based on the distance between data points within the cluster and its centroid, the inter-cluster distance is determined by the distance between the cluster and its farthest neighbour. The COP index is defined by the Eq. (20).

20 $$\:COPindex=\:\frac{1}{n}\sum\:_{a=1}^{k}{n}_{a}\frac{\raisebox{1ex}{$1$}\!\left/\:\!\raisebox{-1ex}{${n}_{a}$}\right.\sum\:_{b=1}^{{n}_{a}}d({x}_{a}\:,{x}_{b})}{\underset{{x}_{p}\notin\:{c}_{a}}{\text{min}}\underset{{x}_{q}\notin\:{c}_{a}}{\text{max}}\left\{d({x}_{p}\:,{x}_{q})\right\}}$$

where:

$\:{c}_{a}$ = the a^th^ cluster.

Sym-Index: The Sym-Index estimates the overall average symmetry of clusters with respect to the cluster centroids for measuring the validity of a clustering result^56^. The composition of the Sym-Index is based on three factors: the number of clusters, the total within-cluster symmetry and the maximum separation between a pair of points in the dataset. The Sym-Index function is defined in Eq. (21).

21 $$\:SymIndex=\:\left(\frac{1}{k}\:\times\:\:\frac{1}{\sum\:_{a=1}^{k}{\sum\:}_{b=1}^{{n}_{a}}{d}_{ps}^{*}({x}_{j}^{a},\:{cc}_{a})}\:\times\:\:{max}_{a,b=1}^{k}\:{\parallel}{cc}_{a}-{cc}_{b}{\parallel}\right)$$

SV Index: The SV index evaluates the validity of a clustering result by finding the ratio of cluster cohesion to cluster separation. The cluster cohesion is measured based on the cluster centroid’s distance to the cluster’s border points. In contrast, the cluster separation is estimated based on the nearest neighbour distance^22,24^. The SV-index is defined using Eq. (22).

22 $$\:SVindex=\:\frac{\sum\:_{a=1}^{k}{{min}_{b\in\:\left[1\cdots\:k\right],\:a\ne\:b}d({u}_{a}\:,\:{u}_{b})}_{\:\:}}{\sum\:_{a=1}^{k}{{max}_{{x}_{b\in\:{C}_{b}}}d({x}_{b}\:,\:{u}_{b})}_{\:\:}}$$

Pakhira-Bandyopadhyay Maulik Index: The PBM Index measures the validity of a clustering result by finding the ratio of the total distance between each point and their corresponding cluster centroid (inter-cluster distance) to the total distance between each point and the global centroid^57^. The PBM Index is defined in Eq. (23).

23 $$\:PBMindex=\:{\left(\frac{1}{k}\:\times\:\frac{\sum\:_{b=1}^{n}\:d({x}_{b}\:,{u}_{0}\:)}{\sum\:_{a=1}^{k}\sum\:_{x\in\:{C}_{a}}d(\:x\:,{u}_{a}\:)}\:\times\:\genfrac{}{}{0pt}{}{k}{\genfrac{}{}{0pt}{}{max}{a,b=1}}\:d({u}_{a}\:,{u}_{b}\:)\right)}^{2}$$

Score Function: The Score Function index is based on maximising the between-class distance while minimising the within-class distance. The SF uses the centroid comparison method to measure the distance between two clusters^58^. The SF index is defined using Eq. (24).

24 $$\:SFindex=\:\:1-\:\frac{1}{{e}^{{e}^{intercd-intracd}}}$$

25 $$\:intercd=\:\frac{1}{nk}\sum\:_{a=1}^{k}d{({cc}_{a},{gc}_{tot})}^{2}\:\cdot\:\:{n}_{a}$$

26 $$\:intracd=\:\frac{1}{k}\:\sum\:_{a=1}^{k}\sqrt{\frac{1}{{n}_{a}}}\sum\:_{x\in\:{C}_{a}}d{(x,{cc}_{a})}^{2}$$

SymDB Index: The SymDB index is a point symmetry-distance-based index with a modification of the cluster cohesion estimator of the DB index^22^. SymDB index is a DB index with a modified cluster cohesion estimator. The modified cluster cohesion estimator is given in Eq. (27). The SymDB index is defined using Eq. (28).

27 $$\:{Z}_{a}=\:\raisebox{1ex}{$1$}\!\left/\:\!\raisebox{-1ex}{$\left|c\right|$}\right.\sum\:_{a=1}^{c}{d}_{ps}^{*}\left({x}_{a}\:,\:{cc}_{a}\right)$$

28 $$\:SymmDBIndex=\:\frac{1}{c}\sum\:_{a}^{c}\underset{a\ne\:b}{\text{max}}\left\{\frac{{Z}_{a}+{Z}_{b}}{d({cc}_{a},{cc}_{b})}\right\}$$

Point Symmetry Index: The Point Symmetry index is a function for measuring clustering validity based on the overall average symmetry with respect to the cluster centres. The definition for the Point Symmetry index is given in Eq. (29).

29 $$\:PSindex=\:\frac{1}{c}\sum\:_{a=1}^{c}\left[\frac{1}{{n}_{a}}\sum\:_{b\in\:{Ce}_{a}}\frac{{d}_{c}({x}_{b},{cc}_{b})}{{d}_{min}}\right]$$

30 $$\:{d}_{c}\left({x}_{b},{cc}_{b}\right)=\:\underset{k=1,\:\cdots\:,{n}_{a,}k\ne\:b}{\text{min}}\left\{\frac{{\parallel}\left({x}_{b}-{cc}_{a}\right)+({x}_{k}-{cc}_{a}){\parallel}}{({\parallel}{x}_{b}-{cc}_{a}{\parallel}+{\parallel}{x}_{k}-{cc}_{a}{\parallel})}\right\}$$

SymDunn: The SymDunn index is also a point symmetry-distance-based index with a modification of the cluster cohesion estimator of the Dunn index^22^. The SymDunn index is a Dunn index with a modified cluster cohesion estimator. The modified cluster cohesion estimator is given in Eq. (31). The definition for the SymDunn index is shown in Eq. (32).

31 $$\:\omega\:\left({C}_{k}\right)=max\left\{{d}_{ps}^{*}\left({x}_{a},{x}_{b}\right)\::\:{x}_{a},{x}_{b}\in\:{C}_{k}\right\}$$

32 $$\:SymDunn=\frac{\:{min}_{1\le\:a\le\:c}\left\{\underset{1\le\:b\le\:c;b\ne\:a}{\text{min}}\left\{\phi\:({C}_{a},{C}_{b})\right\}\right\}\:}{\underset{1\le\:k\le\:c}{\text{max}}\left(\omega\:\left({C}_{k}\right)\right)}$$

### Evolutionary K-means algorithm

K-Means is one of the classical algorithms widely used for solving clustering problems in data analysis and applications. In the K-Means algorithm, the user specifies the number of clusters as an initial parameter. The algorithm then selects k-numbered initial cluster centres and partitions the dataset into the specified number of clusters using Euclidean distance as the objective function. New cluster centroids are chosen, a new partition is generated at each iteration until cluster membership is stable, the maximum number of iterations is achieved, and the termination criteria are realised. The basic steps for the traditional K-Means algorithm and the standard pseudocode can be obtained in^10^.

Specifying an initial number of clusters limits its application for handling automatic clustering problems, hence the need for hybridisation with metaheuristic optimisation algorithms. Moreover, this requirement of specifying the number of clusters as an initial parameter a priori incapacitates the adequate performance of K-means clustering when dealing with big data. An automatic clustering problem requires determining a dataset’s optimal number of clusters. Addressing the challenges associated with the traditional K-means algorithm to improve its performance is a significant research area that has been and is still being exploited by researchers.

Several metaheuristic algorithms, such as GA, PSO, FA, SOS, and many others, have been hybridised with K-means to enhance their capability to find solutions to automatic clustering problems. This report evaluates the performance of recently proposed enhanced FA-K-means, which combines an improved version of the K-means algorithm with an enhanced version of the nature-inspired Firefly metaheuristic optimisation algorithm on fifteen different CVIs to find a solution to the automatic clustering problem. The description and illustration of an automatic clustering problem can be found in^10^.

Metaheuristics algorithms are hybridised with K-means to enhance the capability of the latter in solving the automatic clustering problem. The automatic clustering problem is an optimisation problem that optimises a stated objective function for finding the optimal number of clusters and the corresponding natural groupings inherent in the datasets.

### Enhanced FAK-means

The Firefly algorithm is a swarm-based nature-inspired metaheuristic optimisation algorithm that drew inspiration from the natural behaviour of firefly. The natural fireflies communicate among themselves by emitting bioluminescence flashes. These flashes serve as a system for sending communication signals to others. The signalling system includes the flashing rate, intensity, the time interval between flashes, the brightness, and the flash’s timing accuracy. The firefly metaheuristic algorithm uses these signalling characteristics in its optimisation procedure.

The optimisation problem’s solution vector uses the firefly’s movement from point to point. It is designed based on three basic assumptions: the attractiveness and brightness of a firefly are directly proportional to each other, all fireflies are unisex, and the fitness function landscape determines the brightness of a firefly flashlight. The movement of a firefly from one point to another point is illustrated in Eq. (1).

1 $$\:{X}_{i}^{t+1}=\:{X}_{i}^{t}\:+{\beta\:}_{o}{e}^{-\gamma\:{r}_{ij\:}^{2}}\left({X}_{j}^{t}-\:{X}_{i}^{t}\right)+\:\alpha\:{\in\:}_{i}^{t}$$

*such that*:

$$\:{\beta\:}_{o}=\:\text{a}\text{t}\text{t}\text{r}\text{a}\text{c}\text{t}\text{i}\text{v}\text{e}\text{n}\text{e}\text{s}\text{s}\:\text{c}\text{o}\text{n}\text{s}\text{t}\text{a}\text{n}\text{t}\:\text{f}\text{o}\text{r}\:{r}_{ij}=0$$

$\:{r}_{ij}$ = the distance between two fireflies

$\:\propto\:$= scaling factor controlling the random walk step sizes

$\:\alpha\:{\in\:}_{i}^{t}\:$= randomisation term where $\:{\in\:}_{i}^{t}$ is the Gaussian distribution for random values generation at each iteration

$\:{\beta\:}_{o}{e}^{-\gamma\:{r}_{ij\:}^{2}}\left({X}_{j}^{t}-\:{X}_{i}^{t}\right)$ = Non-linear attractiveness of firefly

The brightness of a firefly is affected by the distance and the intensity of the emitted light. It is illustrated in Eq. (2):

2 $$\:I\left(r\right)={I}_{o}{e}^{-\gamma\:{r}^{2}}\:\:$$

*such that*:

$$\:r\:$$= distance

$\:{I}_{o}\:$= intensity of light at $\:r=0$

$\:\gamma\:$ = coefficient of light absorption

Where the distance between two fireflies is illustrated in Eq. (3):

3 $$\:{r}_{ij}=\:{\parallel}{x}_{i}-\:{x}_{i}{\parallel}=\:\sqrt{\sum\:_{k=1}^{d}{\left({x}_{i,k}-\:{x}_{j,k}\right)}^{2\:}}$$

#### Such that

$\:d$= dimension of the problem.

Enhanced FA-K-means is one of the evolutionary K-means algorithms recently proposed to enhance K-means algorithm performance for handling automatic clustering problems. The enhanced FA-K-means^10^ combined CLT-based K-means with an enhanced firefly algorithm. The optimum number of clusters is automatically generated in the hybridised algorithm, and the initial cluster centroids are determined. The enhanced K-means use these parameters to solve the clustering problem. The full description of the enhanced FA-K-means and the CLT-based K-means with the associated algorithms can be found in^10^.

The clustering performance of the Enhanced FA-K-means has been reported as superior to other contemporary K-means-based metaheuristics such as the SOSK-means^9^ and Improved SOSK-means^8^ for automatic clustering. Metaheuristic-based automatic clustering techniques represent an advanced paradigm in unsupervised learning, combining global exploration (via metaheuristics) with local refinement (through embedded clustering algorithms). Our Enhanced Firefly-K-means (EFKM) algorithm embodies this approach by dual optimization mechanism via global search - exploring the solution space to avoid local optima in cluster assignments and local refinement with the K-means fine-tuning the cluster centroids for precise boundary determination.

The algorithm dynamically identifies the optimal cluster count through multi-objective optimization of.

minimizing the intra-cluster compactness and maximizing the inter-cluster separation to ensure solution stability across iterations. Unlike the static approaches, the algorithm exhibits theoretical advantage over conventional methods through simultaneous optimization of cluster quality; adapting to dataset-specific characteristics through its stochastic search process thereby achieving robust performance regardless of initial conditions.

An initial population of fireflies is generated at the commencement of the search process where each member of the population represents a candidate solution. The objective (fitness) function is evaluated using the cluster validity index to determine the fitness score of each of the candidate solution and the candidation solution with the best score is retained. Iteratively, new solutions that produces better fitness score are subsequently updated using the firefly operators. The final solution obtained from the evolutionary optimisation phase is then passes to the CLTbased K-means as the initial cluster centroids. The run cycle involving the two phases complete the execution of the algorithm and the best solution at each run cycle is chosen as the optimal value. The pseudocode for the FA-K-means algorithm is presented in Algorithm 1.

**Algorithm 1:**
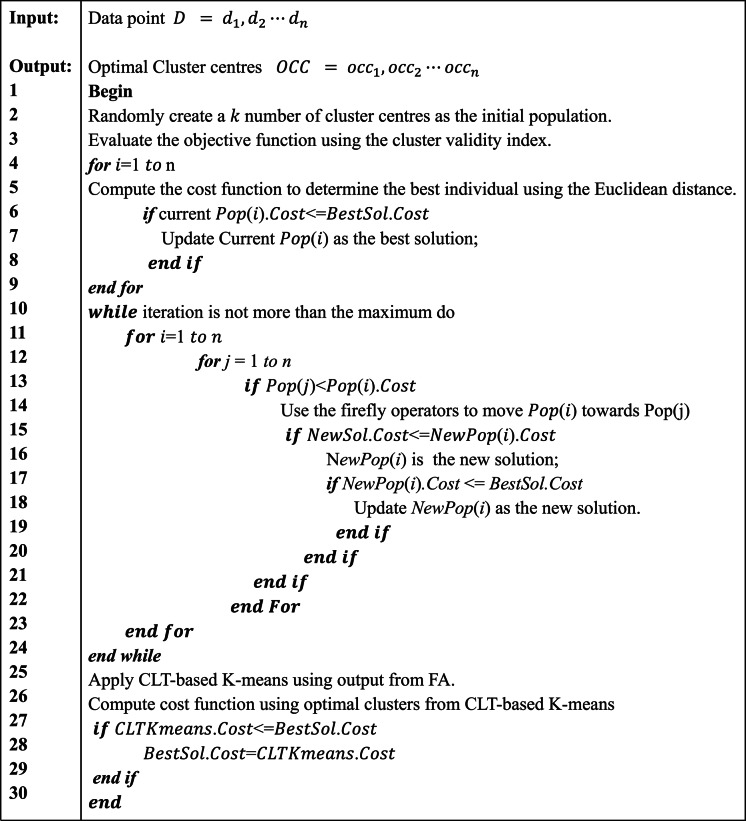
Pseudocode for the FA-K-means Algorithm^10^.

The Pop(i).Cost is an array of firefly structures, each firefly representing a potential solution with pop(i) representing the *i-th* firefly in the population and the pop(i).Cost is the cost (fitness) value of that solution based on the CVI used. The BestSol is a structure that stores the best solution found so far during the optimization process. It is the lowest cost (fitness value) among all fireflies seen so far, the best clustering configuration based on the index. It is used to monitor and save the global best solution. It guides the search towards the optimal solution.

## Experimental study, performance evaluation and discussion

This section reports the experimental study and the performance evaluation of the different cluster validity indices on twelve datasets with varied characteristics using the Enhanced FA-K-means clustering algorithm. It presents details on the other datasets and their characteristics, the Enhanced FA-K-means parameter settings, and the various CVIs’ performance scores on the multiple datasets.

### Performance measures

The experimental study involves fifteen distinct CVIs applied to datasets with diverse characteristics to assess their effectiveness as fitness functions for the Enhanced Firefly-K-means algorithm, an evolutionary K-means automatic clustering algorithm. The performances of the CVIs were measured in terms of their success rates and computation time. The primary objective of the experiment is to analyse the performance of these CVIs on the evolutionary K-means framework to identify which index performs optimally for each dataset exhibiting specific characteristics in the context of automatic clustering. The following criteria were considered for the evaluation:

i. Clustering solution quality for the different CVIs based on the average best fitness value.
ii. Computational cost to determine the performance speed.
iii. Statistical Analysis of the CVIs Performance.

### Parameter setting and system configuration

The parameter setting for the Enhanced Firefly-K-means clustering algorithm is summarised in Table 1. It presents the initial parameter values for the FA. For each CVI, the FAK-means algorithm is executed based on a maximum function evaluation of 50,000 with a swarm size of 40 + 2k. The execution is replicated 20 times for all the 12 datasets. An Intel Dual Corei7-7600U CPU computer machine with 15.8 GB RAM and 2.80 GHz CPU speed with MATLAB 2018 was installed and used for the simulation experiment. The experimental conditions apply to all the tested experimental instances in this paper.

**Table 1:** Fak-Means algorithm parameter Setting.

Parameter	Description	Value
MaxFE	Maximum Function Evaluation	50,000
NP	Population Size	40 + 2k
m	Mutation Coefficient	2
$\:\beta\:$	Attraction Coefficient	2
$\:\gamma\:$	Coefficient for Light Absorption	1
$\:\propto\:$	Damping Ratio	1

### Description of datasets

Both real-life and synthetic datasets with varying characteristics were used in the experiment. The real-life datasets include glass, thyroid, yeast, breast, iris, and wine, which are representations of science and engineering datasets. These are commonly used datasets in clustering performance evaluation. The synthetic datasets include Compound and Flame, representing datasets with non-linear separable clusters; Spiral, Path-based, and Two-moon, representing datasets having arbitrarily shaped with overlapping clusters; and Jain, representing clusters with more complex paths. The mean of the best fitness scores over twenty iterations for each dataset for the different CVIs are used in the study. Table 2 contains the summary of the twelve datasets and their characteristics. The description and equations of the CVIs used in the experiment have been presented earlier in this paper. The visual impressions of the performance of the clustering outputs on the various datasets based on the best three performing CVIs are given in Appendix 1.

**Table 2:** Characteristics of the Real-Life and synthetic Datasets.

Type of dataset	Dataset characteristics	Name of dataset	Number of features	Number of 0bjects	Number of clusters	References
Real-life	UCI	Glass	9	214	6	^59,60^
		Thyroid	5	215	2	^59,60^
		Yeast	8	1484	10	^59,60^
		Breast	9	699	2	^59,60^
		Iris	4	150	3	^59,60^
		Wine	13	178	3	^59,60^
Synthetic	Non-linearly Separable Clusters	Flame	2	240	2	^59,61^
		Compound	2	399	6	^59,62^
	arbitrarily overlapping shaped clusters	Spiral	2	312	2	^59,63^
		Path-based	2	300	3	^59,63^
		Two-Moons	2	10,000	2	
	With complex path clusters	Jain	2	373	2	^59,64^

### Performance evaluation and discussion

**Fig. 1:**
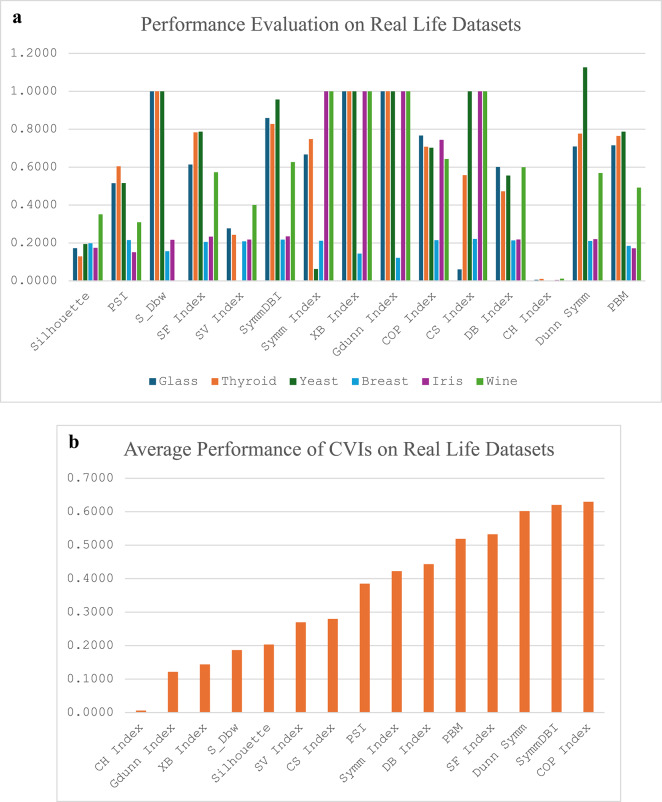
**a**. Performance report of CVIs on real life datasets **b**. Average performance report of CVIs across real life datasets.

We present the report of the average performances of the fifteen different CVIs as the objective function for the FA-K-means algorithm in Table 3. All the CVIs were evaluated as minimisation functions for fair comparison. The CVIs that are naturally maximisation functions were mathematically manipulated to minimise their functions (PBM, Silhouette, PSI, SF index, SV index, G-Dunn, Symm index, CH index and Dunn-Symm). Euclidean distance was used as the default metric across all experiments for direct comparability with prior CVI benchmarking studies. This choice aligns with the assumptions of most internal CVIs (e.g., Davies-Bouldin, Silhouette). All preprocessing steps and distance metrics were fixed across datasets unless explicitly stated (e.g., in robustness tests where noise/scale variations were introduced).We present the average performance report of CVIs of twenty runs for each of the different groupings of the datasets – the real-life dataset and the synthetic dataset. The synthetic data sets are further subdivided into three groups – those with non-linearly separable clusters, those with arbitrarily overlapping shape clusters and those with complex path clusters. All datasets were standardized using Z-score normalization (mean = 0, variance = 1) to ensure features were on comparable scales, mitigating bias toward high-magnitude dimensions. In Fig. 1a, the performance evaluation of the CVIs on the various real-life datasets is presented as a clustered column chart, and their mean performance across the datasets is presented as a column chart in Fig. 1b.

**Table 3:** Average clustering performance of the CVIs on each Dataset.

	Real-life datasets						Synthetic datasets					
	UCI						Non-linearly separable clusters		Arbitrarily overlapping shaped clusters			Complex path clusters
	Glass	Thyroid	Yeast	Breast	Iris	Wine	Flame	Compound	Spiral	Path-based	Two-moons	Jain
Silhouette	0.1725	0.1287	0.1944	0.1982	0.1745	0.3511	0.3095	0.1174	0.3272	0.2299	0.2428	0.2510
PSI	0.5148	0.6043	0.5158	0.2152	0.1514	0.3094	0.6424	0.0906	0.8565	0.6107	0.2855	0.2301
S_Dbw	1.0000	1.0000	1.0000	0.1562	0.2167	1.0000	1.0000	0.1227	1.0000	0.4746	0.3021	0.2880
SF Index	0.6140	0.7834	0.7870	0.2052	0.2333	0.5728	0.5723	0.1323	0.6367	0.2995	0.3202	0.2984
SV Index	0.2770	0.2431	1.0000	0.2085	0.2179	0.4008	0.3868	0.1165	0.3978	0.3335	0.2964	0.2952
SymmDBI	0.8591	0.8277	0.9567	0.2175	0.2345	0.6266	0.8483	0.1264	0.9510	0.5688	0.3203	0.3117
Symm Index	0.6668	0.7480	0.0627	0.2116	1.0000	1.0000	0.7404	1.8489	1.8400	0.0269	0.3270	2.2095
XB Index	1.0000	1.0000	1.0000	0.1438	1.0000	1.0000	1.0000	0.0874	0.0000	0.5778	1.0000	1.0000
Gdunn Index	1.0000	1.0000	1.0000	0.1216	1.0000	1.0000	1.0000	0.1264	0.0000	0.5546	1.0000	1.0000
COP Index	0.7665	0.7080	0.7025	0.2148	0.7439	0.6429	0.7439	0.1320	0.9271	0.5732	0.3174	0.3081
CS Index	0.0608	0.5578	1.0000	0.2212	1.0000	1.0000	1.0000	0.1209	0.0000	0.5298	1.0000	0.0608
DB Index	0.6009	0.4725	0.5559	0.2132	0.2183	0.5995	0.7428	0.1311	0.7858	0.5684	0.3197	0.3112
CH Index	**0.0057**	**0.0105**	**0.0023**	**0.0010**	**0.0040**	**0.0119**	**0.0063**	**0.0010**	**0.0052**	**0.0027**	**0.0006**	**0.0020**
Dunn Symm	0.7088	0.7771	1.1261	0.2103	0.2197	0.5687	0.7388	0.1276	0.9060	0.5337	0.3087	0.2954
PBM	0.7150	0.7644	0.7870	0.1844	0.1718	0.4917	0.6025	0.1275	0.6367	0.2995	0.3070	0.2880

The CH index recorded the best performance scores for the real-life dataset, while the COP index recorded the worst performance for all the real-life datasets. While CH, Silhouette, PSI, SF index, Sym-DBI, DB index, CH index, Dunn Sym, PBM and COP could cluster all the datasets, the other CVIs showed some difficulty in handling some of the datasets. For instance, the SV index had difficulty in clustering the Yeast dataset; S_Dbw was only able to cluster Breast and Iris datasets; the Symm index clustered Glass, Thyroid, Yeast and Breast datasets; the CS index clustered Glass, Thyroid and Breast datasets; the XB index and G-Dunn index were able to cluster only Breast datasets successfully.

All the CVIs could comfortably cluster the Breast dataset, where, in most cases, they recorded their best performance except for PSI, CSI, Symm index, PBM, and Silhouette. The Silhouette, PSI, CS index and PBM recorded their best performances on Thyroid, Yeast, and Glass, respectively, while PSI and PBM had their best performance on the Iris dataset. The SF, Sym-DBI, Dunn Sym and PBM had their worst performance on the Yeast dataset; the PSI, Symm, and CS recorded their worst performance on Thyroid; COP and DB had their worst performance on Glass while Silhouette, S_Dbw and SV indices have their worst performance on Breast, Iris and Wine respectively.

The synthetic datasets have three categories: the non-linearly separable cluster (Flame and Compound), the arbitrary overlapping shape clusters (Spiral, Path-based, Two-moons) and the Complex path clusters (Jain). For the synthetic datasets, most of the CVIs except XB, G-Dunn and CS indices were able to cluster all the groups successfully. While S_Dbw could cluster most of the synthetic datasets except Flame and Sprial, the CS index was only able to cluster one in each of the groups (Compound, Path-based and Jain), XB and G-Dunn could only cluster Compound and Path-based datasets. Most CVIs recorded their best performances on the Compound datasets except for CS and Symm indexes. The CS and Symm indexes perform best on the Jain and Path-based datasets. It is worth noting that while other CVIs have the best performance on the Compound dataset, the Symm-index recorded its worst performance on the Compound dataset. Also, among all the other CVIs, only the CS index performed exceptionally well on the Jain dataset, representing a dataset with complex path clusters. Seven of the CVIs recorded their worst performance on the Spiral datasets (Silhouette, PSI, SF, SV, Symm-DB, DB and PBM); four out of the remaining six CVIs recorded their worst performance on Path-based datasets (S_Dbw, XB, G-Dunn and CS); while the COP and Dunn-Sym recorded their worst performances on Flame dataset. For all the categories of the synthetic datasets, the CH index is credited with the best performance scores compared with all the other CVIs, with its best and worst scores being better than the others.

**Fig. 2:**
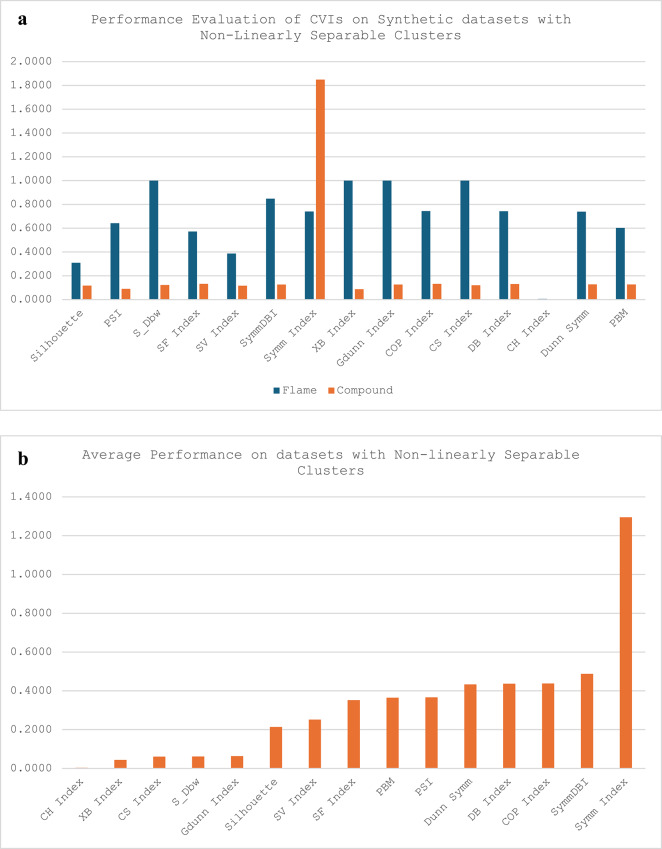
**a**. Performance report of CVIs on synthetic datasets with non-linearly separable clusters **b**. Average performance report of CVIs on datasets with non-linearly separable clusters.

**Fig. 3:**
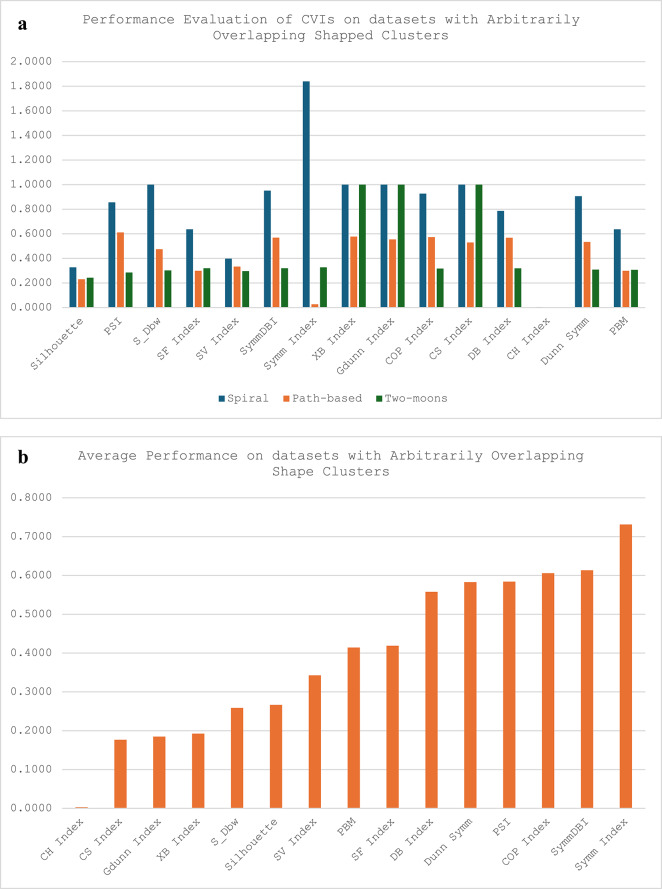
**a**. Performance report of CVIs on synthetic datasets with arbitrarily overlapping shape clusters **b**. Average performance of CVIs on datasets with arbitrarily overlapping shape clusters.

**Fig. 4:**
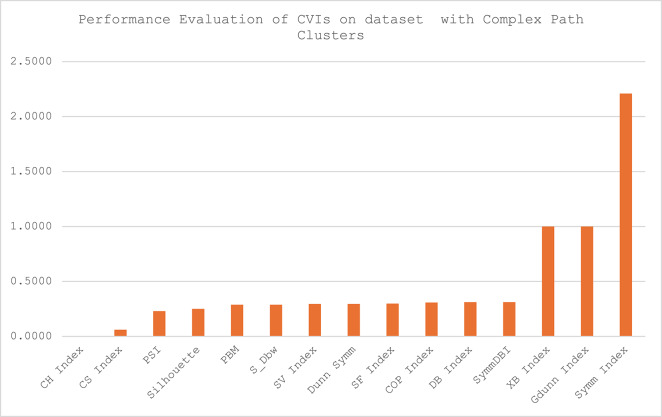
Performance report of CVIs on synthetic datasets with complex path clusters.

The performances of each CVI for each category of the synthetic datasets are presented as column charts in Figs. 2a, 3a and 4. Figure 2a shows that all the CVIs except the Symm index recorded excellent clustering performances on the Compound dataset with scores better than the average performance across the CVIs. On the other hand, seven out of eleven of the CVIs that were able to cluster the Flame dataset recorded scores worse than the average performance recorded across the CVIs.

Figure 2b presents the average performance report of the CVIs across the datasets characterised with non-linearly separable clusters, the Flame and Compound datasets. It can be observed that the CH index recorded the best average performance scores across the two datasets, while the Symm index has the worst average performance scores. 

Figure 3a shows that all the CVIs were able to cluster the Path-based datasets. However, for the other two datasets in this category, S_Dbw, XB index, GDunn and CS index could not produce a reasonable result on the Spiral dataset, while XB index, GDunn and CS index were not able to handle the Two-moon dataset. For this category of synthetic datasets, the CH index has the best average performance score, while the Symm index recorded the worst performance score, as shown in Fig. 3b.

The performance report of the CVIs on synthetic datasets with complex path clusters presented in Fig. 4 shows that the CH index outperformed all the other CVIs. It can be observed that only two of the CVIs - the XB index and the GDunn index could not produce a conclusive result in handling the Jain datasets. It is also worth noting that Symm Index performance was poor in clustering datasets with complex path clusters.

The performance report of the CVIs on synthetic datasets with complex path clusters presented in Fig. 4 shows that the CH index outperformed all the other CVIs. It can be observed that only two of the CVIs - the XB index and the GDunn index could not produce a conclusive result in handling the Jain datasets. It is also worth noting that Symm Index performance was poor in clustering datasets with complex path clusters.

**Table 4:** Average computation time of the CVIs on each dataset.

	Real-life datasets						Synthetic datasets					
	UCI						Non-linearly separable clusters		Arbitrarily overlapping shaped clusters			Complex path clusters
	Glass	Thyroid	Yeast	Breast	Iris	Wine	Flame	Compound	Spiral	Path-based	Two-moons	Jain
Silhouette	21.63	22.24	106.61	106.39	21.14	31.17	45.64	22.54	39.27	38.84	78.75	31.01
PSI	18.16	20.65	23.42	17.62	19.66	22.34	17.88	17.47	20.24	19.41	20.80	19.92
S_Dbw	124.41	123.83	347.68	195.38	134.35	143.52	129.89	140.89	135.35	207.78	167.41	141.81
SF Index	107.53	109.69	172.65	131.78	108.30	113.39	108.54	113.58	112.85	134.60	126.07	113.87
SV Index	93.44	92.50	163.32	108.27	91.93	96.06	92.64	96.03	94.41	100.79	102.56	94.26
Sym-DBI	15.46	17.01	20.34	17.02	16.80	17.46	15.75	15.04	16.20	16.67	16.79	16.03
Symm Index	17.00	19.03	26.16	35.22	18.10	20.40	16.96	16.81	18.56	20.26	19.18	17.45
XB Index	18.80	21.70	27.44	27.01	20.06	22.26	18.52	18.25	20.29	19.77	21.70	19.68
Gdunn Index	26.57	25.51	48.31	18.65	28.47	29.91	33.97	19.06	30.72	23.80	41.51	25.41
COP Index	20.58	20.93	21.65	18.70	19.77	20.31	19.77	19.10	20.51	19.88	20.68	20.00
CS Index	23.56	31.39	93.97	56.37	25.67	36.40	30.26	25.19	46.17	66.82	79.07	23.56
DB Index	108.66	107.44	414.95	132.23	118.12	109.77	106.98	111.74	111.32	131.46	119.11	130.21
CH Index	22.58	26.03	39.39	53.53	24.53	24.58	22.84	25.64	24.37	23.25	23.96	23.34
Dunn Symm	799.65	124.27	91.67	117.71	130.40	55.00	210.92	225.09	62.58	252.78	361.86	96.88
PBM	45.22	45.92	71.92	53.63	45.60	45.69	45.42	44.58	45.46	53.58	53.92	45.34

**Fig. 5:**
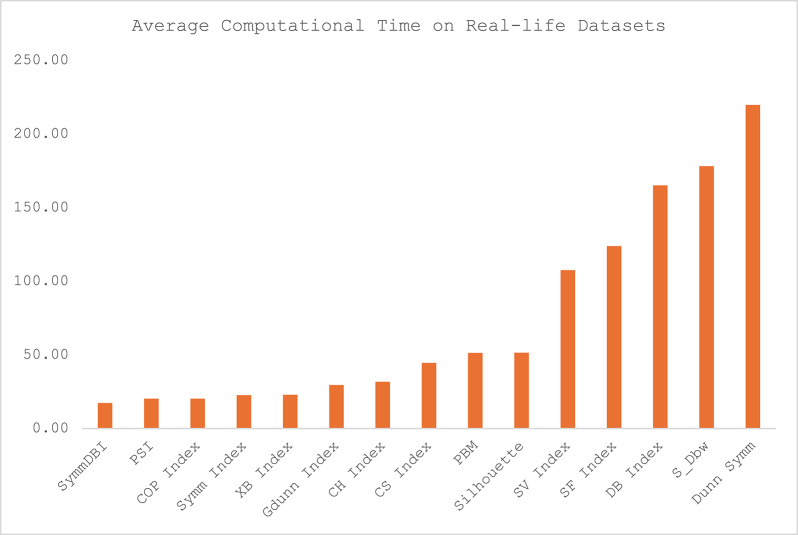
Average computational time on real-life datasets.

**Fig. 6:**
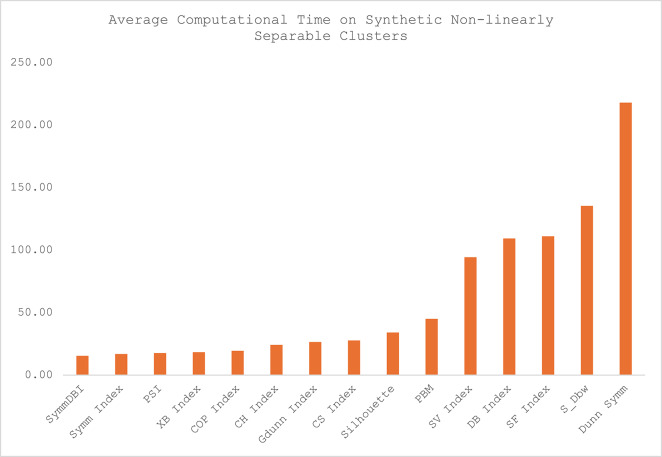
Average computational time on synthetic datasets with non-linearly separable clusters.

**Fig. 7:**
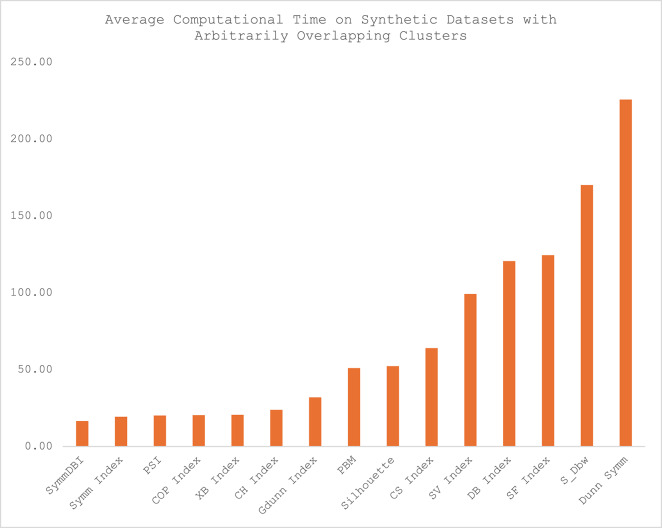
Is a bar chart showing the average computational time on real-life datasets.

**Fig. 8:**
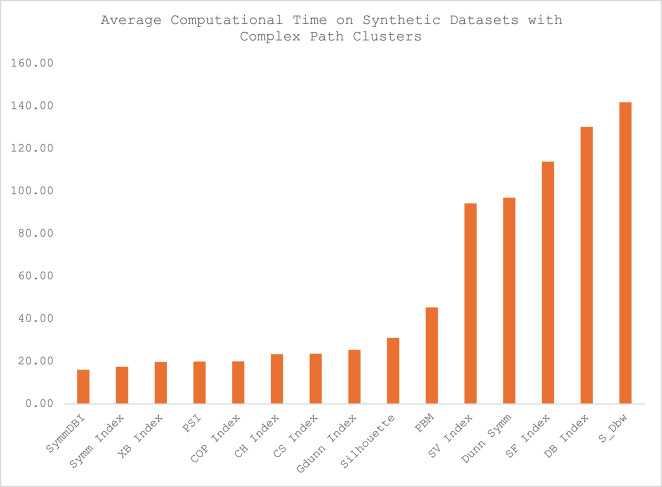
Average computational time on synthetic datasets with complex path clusters.

The average computational time for each of the CVIs across the twelve datasets is presented in Table 4, and the illustration of the computational time for each category of the datasets is presented in Figs. 5, 6, 7 and 8. Sym-DBI has the lowest computational time across the various categories of the dataset. At the same time, the Dunn Symm consumed the largest computational time for all the categories except for the synthetic datasets with complex data paths, where S_Dbw recorded the highest computational time. The computational time for the Symm index, PSI, COP, XB and CH index are relatively low compared with the other CVIs.

**Fig. 9:**
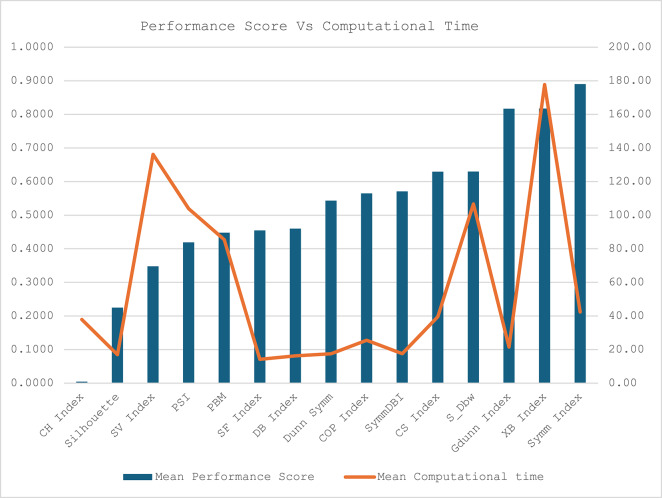
Comparing CVIs performance scores and computational time across the datasets.

The average performance score and computational time for each CVI across the twelve datasets is presented in Table 5. While the CH index has the lowest mean performance score, the DB index recorded the smallest computational time. The Symm index has the worst performance score, while the XB recorded the worst computational time. The Silhouette index demonstrated excellent performance in fitness score and computational time compared with SV, PSI, and PBM, with a good performance score at the expense of high computational time. The average computational time for SF, DB, Dunn Symm, COP and Sym DB are relatively low, but the performance scores are relatively high compared with the CH index and Silhouette. The comparison between the mean performance scores and computational time for all the CVIs is illustrated in Fig. 9 for better visualisation.

**Table 5:** Comparison between the mean performance score and mean computational time.

Cluster Validity Index	Mean Performance Score	Mean Computational time
CH Index	0.0044	37.90
Silhouette	0.2248	16.97
SV Index	0.3478	136.19
PSI	0.4189	103.88
PBM	0.4480	85.42
SF Index	0.4546	14.21
DB Index	0.4599	16.26
Dunn Symm	0.5434	17.51
COP Index	0.5650	25.54
SymmDBI	0.5707	17.58
CS Index	0.6293	39.57
S_Dbw	0.6300	106.66
Gdunn Index	0.8169	21.51
XB Index	0.8174	177.71
Symm Index	0.8902	42.36

### Statistical analysis

**Fig. 10:**
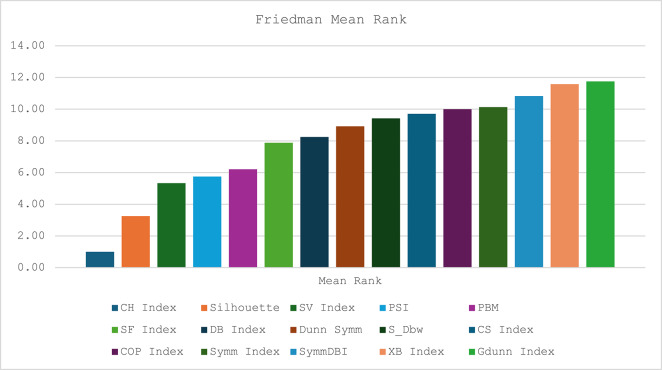
Friedman mean rank test analysis.

We subjected the experiment results obtained to statistical analysis for validation. We conducted a non-parametric Friedman’s rank test, like ANOVA, to determine if there is any significant difference between each of the different CVIs across the twelve datasets. This test is applied to each of the datasets at a p-value of 0.05 for the CVIs, and the results are shown in Table 6. The CVI with the smallest mean rank value performs better across the datasets. The CS index has the lowest mean rank across the datasets, followed by the Silhouette index. The worst performance is recorded against the GDunn index, with a mean rank of 11.75. The pictorial illustration for Friedman’s mean rank for all the CVIs is presented in Fig. 10. The Friedman’s test statistics produced a Chi-square value of 82.776 and a p-value of < 0.05. This implies a statistically significant difference in the performance (ranking) of at least one CVI across the 12 datasets.

**Table 6:** Friedman’s rank test for all CVIs.

	Glass	Thyroid	Yeast	Breast	Iris	Wine	Flame	Compound	Spiral	Path-based	Two-moons	Jain	Mean Rank
Silhouette	3.00	2.00	5.00	6.00	4.00	12.00	10.00	1.00	11.00	7.00	8.00	9.00	3.25
PSI	7.00	9.00	8.00	3.00	2.00	6.00	11.00	1.00	12.00	10.00	5.00	4.00	5.75
S_Dbw	9.50	9.50	9.50	2.00	3.00	9.50	9.50	1.00	9.50	6.00	5.00	4.00	9.42
SF Index	9.00	11.00	12.00	2.00	3.00	8.00	7.00	1.00	10.00	5.00	6.00	4.00	7.88
SV Index	5.00	4.00	12.00	2.00	3.00	11.00	9.00	1.00	10.00	8.00	7.00	6.00	5.33
SymmDBI	10.00	8.00	12.00	2.00	3.00	7.00	9.00	1.00	11.00	6.00	5.00	4.00	10.83
Symm Index	5.00	7.00	2.00	3.00	8.50	8.50	6.00	11.00	10.00	1.00	4.00	12.00	10.13
XB Index	8.00	8.00	8.00	2.00	8.00	8.00	8.00	1.00	8.00	3.00	8.00	8.00	11.58
Gdunn Index	8.00	8.00	8.00	1.00	8.00	8.00	8.00	2.00	8.00	3.00	8.00	8.00	11.75
COP Index	11.00	8.00	7.00	2.00	9.50	6.00	9.50	1.00	12.00	5.00	4.00	3.00	10.00
CS Index	1.50	6.00	9.50	4.00	9.50	9.50	9.50	3.00	9.50	5.00	9.50	1.50	9.71
DB Index	10.00	6.00	7.00	2.00	3.00	9.00	11.00	1.00	12.00	8.00	5.00	4.00	8.25
CH Index	9.00	11.00	5.00	2.00	7.00	12.00	10.00	3.00	8.00	6.00	1.00	4.00	1.00
Dunn Symm	8.00	10.00	12.00	2.00	3.00	7.00	9.00	1.00	11.00	6.00	5.00	4.00	8.92
PBM	10.00	11.00	12.00	3.00	2.00	7.00	8.00	1.00	9.00	5.00	6.00	4.00	6.21

**Fig. 11:**
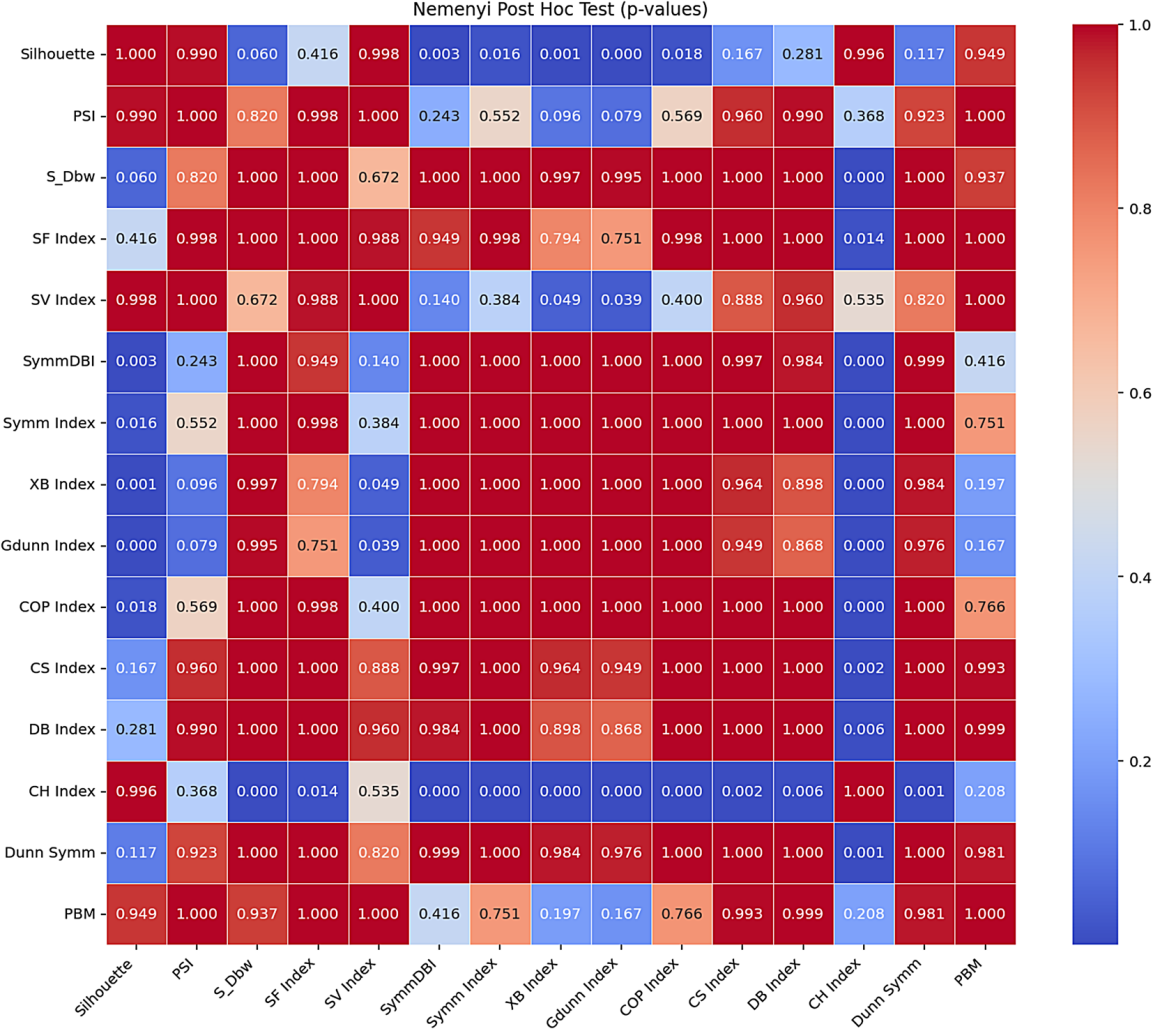
Nemenyi post hoc heatmap.

For further verification of the significant difference in the performance of the CVIs across the datasets as observed in Friedman’s statistics, a post hoc test with Nemenyi was conducted on the CVIs results. The Nemenyi post hoc test presents a set of p-values that statistically establish the significant differences between the various CVIs. The result of the Nemenyi post hoc is shown in Table 7, and the Nemenyi post hoc heatmap shown in Fig. 11 provides a visualisation of the pairwise p-values for comparing the clustering validity indices.

**Table 7:** Nemenyi’s post hoc test for all CVIs.

	Silhouette	PSI	S_Dbw	SF Index	SV Index	Sym DBI	Symm Index	XB Index	Gdunn Index	COP Index	CS Index	DB Index	CH Index	Dunn Symm	PBM
Silhouette	1	0.9899	0.0601	0.4162	0.9984	0.0027	0.0164	0.0005	0.0004	0.0178	0.1666	0.2813	0.9965	0.1166	0.9492
PSI	0.9899	1	0.8204	0.9981	1	0.2427	0.5521	0.0964	0.0791	0.5694	0.9596	0.9899	0.3684	0.9227	1
S_Dbw	0.0601	0.8204	1	1	0.6716	1	1	0.9971	0.9949	1	1	1	0.0005	1	0.9369
SF Index	0.4162	0.9981	1	1	0.9882	0.9492	0.9976	0.7939	0.7507	0.9981	1	1	0.0139	1	0.9999
SV Index	0.9984	1	0.6716	0.9882	1	0.1399	0.3841	0.0486	0.0389	0.4	0.8881	0.9596	0.5348	0.8204	1
SymDBI	0.0027	0.2427	1	0.9492	0.1399	1	1	1	1	1	0.9971	0.984	0	0.9992	0.4162
Symm Index	0.0164	0.5521	1	0.9976	0.3841	1	1	1	0.9999	1	1	0.9997	0.0001	1	0.7507
XB Index	0.0005	0.0964	0.9971	0.7939	0.0486	1	1	1	1	1	0.9642	0.8975	0	0.984	0.1968
Gdunn Index	0.0004	0.0791	0.9949	0.7507	0.0389	1	0.9999	1	1	0.9999	0.9492	0.8676	0	0.9756	0.1666
COP Index	0.0178	0.5694	1	0.9981	0.4	1	1	1	0.9999	1	1	0.9998	0.0001	1	0.7655
CS Index	0.1666	0.9596	1	1	0.8881	0.9971	1	0.9642	0.9492	1	1	1	0.0025	1	0.9927
DB Index	0.2813	0.9899	1	1	0.9596	0.984	0.9997	0.8975	0.8676	0.9998	1	1	0.0063	1	0.999
CH Index	0.9965	0.3684	0.0005	0.0139	0.5348	0	0.0001	0	0	0.0001	0.0025	0.0063	1	0.0014	0.2076
Dunn Symm	0.1166	0.9227	1	1	0.8204	0.9992	1	0.984	0.9756	1	1	1	0.0014	1	0.9815
PBM	0.9492	1	0.9369	0.9999	1	0.4162	0.7507	0.1968	0.1666	0.7655	0.9927	0.999	0.2076	0.9815	1

The heatmap uses colour intensity to illustrate the significance of the differences in the performances of the CVIs. The blue shades represent small p-values indicating strong evidence of differences, and the red shades represent large p-values indicating no significant differences. From the Nemenyi test result, a p-value < 0.05 implies a statistically significant difference between the two corresponding indices. Significant differences between Silhouette and Sym-DBI, Symm Index, XB index, and G-Dunn index can be observed. There are also significant differences between the CH index and most others.

## Conclusions

This study presented a comparative performance evaluation of fifteen different internal cluster validity indices on an evolutionary K-means algorithms framework. Evolutionary K-means algorithms adopt internal CVIs as the objective function for determining the optimal number of clusters and automatically finding the inherent patterns within the dataset. The study’s primary goal is to check which of the CVIs performs best for the different categories of datasets when solving automatic clustering problems using the evolutionary K-means framework. Selecting the appropriate cluster validity index is one of the implementation problems because CVIs are designed to address specific data types, making their performance data dependent. Fifteen internal cluster validity indices were evaluated and compared based on their performances and computational time spent automatically clustering twelve datasets with distinctive characteristics. Both real-life (as representative datasets for science and engineering) and synthetic (as representative datasets with varying characteristics, such as those with non-linear separable clusters, those with arbitrarily shaped overlapping clusters, or those with clusters with more complex paths) datasets were considered.

The improved Firefly-K-Means algorithm served as the evolutionary K-means algorithm framework for the study, and the fifteen CVIs used in the study include DB, CH, CS, Dunn Index, G-Dunn, S_Dbw SIL, XB, COP, Sym-Index, SV, PBM, SF, Sym-DB, and PSI. The real-life datasets included Glass, Thyroid, Yeast, Breast, Iris and Wine; the synthetic datasets with non-linearly separable clusters include the Flame and Compound; the synthetic datasets with arbitrarily overlapping shaped clusters include the Spiral, Path-based and Two-moon, while the Jain dataset represented the dataset with complex path clusters From the experimental study, it can be observed that CH index recorded the best performance score across the entire categories of the dataset. While CH, Silhouette, PSI, SF index, Sym-DBI, DB index, CH index, Dunn Sym, PBM and COP could cluster all the datasets, others had issues clustering one or more. In terms of the computational time, the DB index recorded the smallest computational time while the XB recorded the worst computational time. The Silhouette index demonstrated excellent performance in fitness score and computational time compared with SV, PSI, and PBM, which had good performance scores at the expense of high computational time. The average computational time for SF, DB, Dunn Symm, COP and Sym DB are relatively low, but the performance scores are relatively high compared with the CH index and Silhouette.

The numerical results were subjected to statistical analysis using Friedman’s test to validate the performance scores obtained from the study, which statistically confirms the significant differences in the performance scores of the different CVIs. The Nemenyi post hoc test was also conducted to identify the important differences between various CVIs. Overall, the result of the experimental study shows that the CH and Silhouette indices have the best average performance across entire datasets in terms of fitness scores and computational time, respectively. In line with the observations from existing articles about cluster validity indices, the experimental results show that no single CVI is superior to others, and the observed performances were subject to the dataset’s structure. However, it is advisable to use CH and Silhouette indices when handling automatic clustering tasks to validate optimal clustering solutions using the evolutionary K-means framework based on their uniform and consistently superior performance across the different categories of datasets. For future research, the real-life application of automatic clustering using evolutionary K-means algorithms with identified optimal-performing CVIs can be investigated by clustering enthusiasts.

## Electronic supplementary material

Below is the link to the electronic supplementary material.

Supplementary Material 1

Supplementary Material 2

## Data Availability

All data generated or analysed during this study are included in this article.

## Abbreviations

CVIs: Cluster validity indices
CHI: Calinski-harabasz index
DBI: Davies bouldin index
CSI: Compact separated index
DI: Dunn index
GDI: General dunn index
SI: Silhouette Index
XBI: Xie-beni index
Sym: Index symmetric index
PBM: Pakhira-bandopadhyah maulik index
SF: Score Function Index
SymDB: Symmetric DB index
PSI: Point symmetry index
