# Supplementary figures and images for: Benchmarking validity indices for evolutionary K-means clustering performance
Source: Sci Rep. 2025 Jul 1;15:21842. doi: 10.1038/s41598-025-08473-6 (PMC12218181; doi:10.1038/s41598-025-08473-6)

**Appendix 1:** Clustering Results for twelve datasets based on CH index, Silhouette and SV index

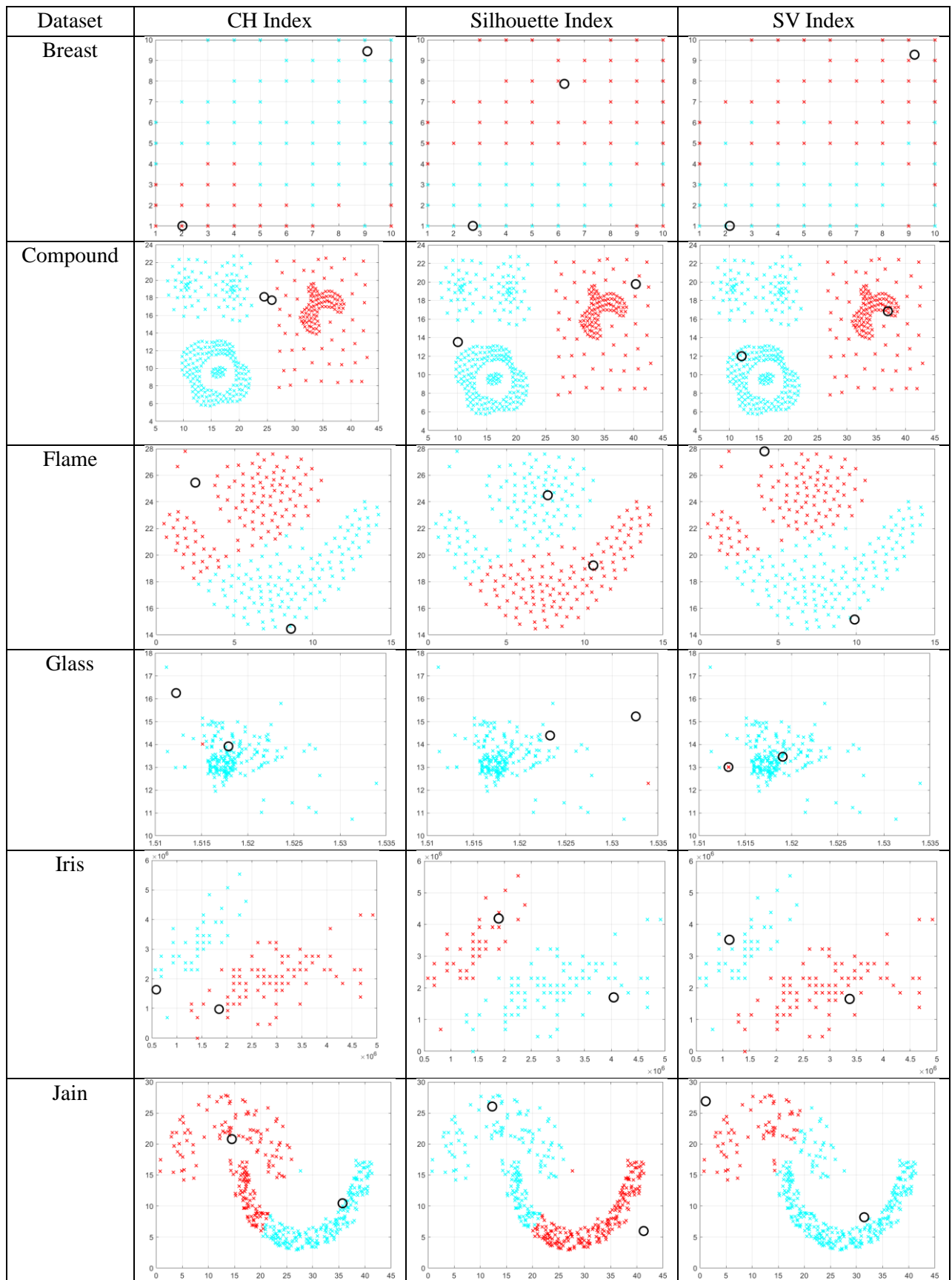

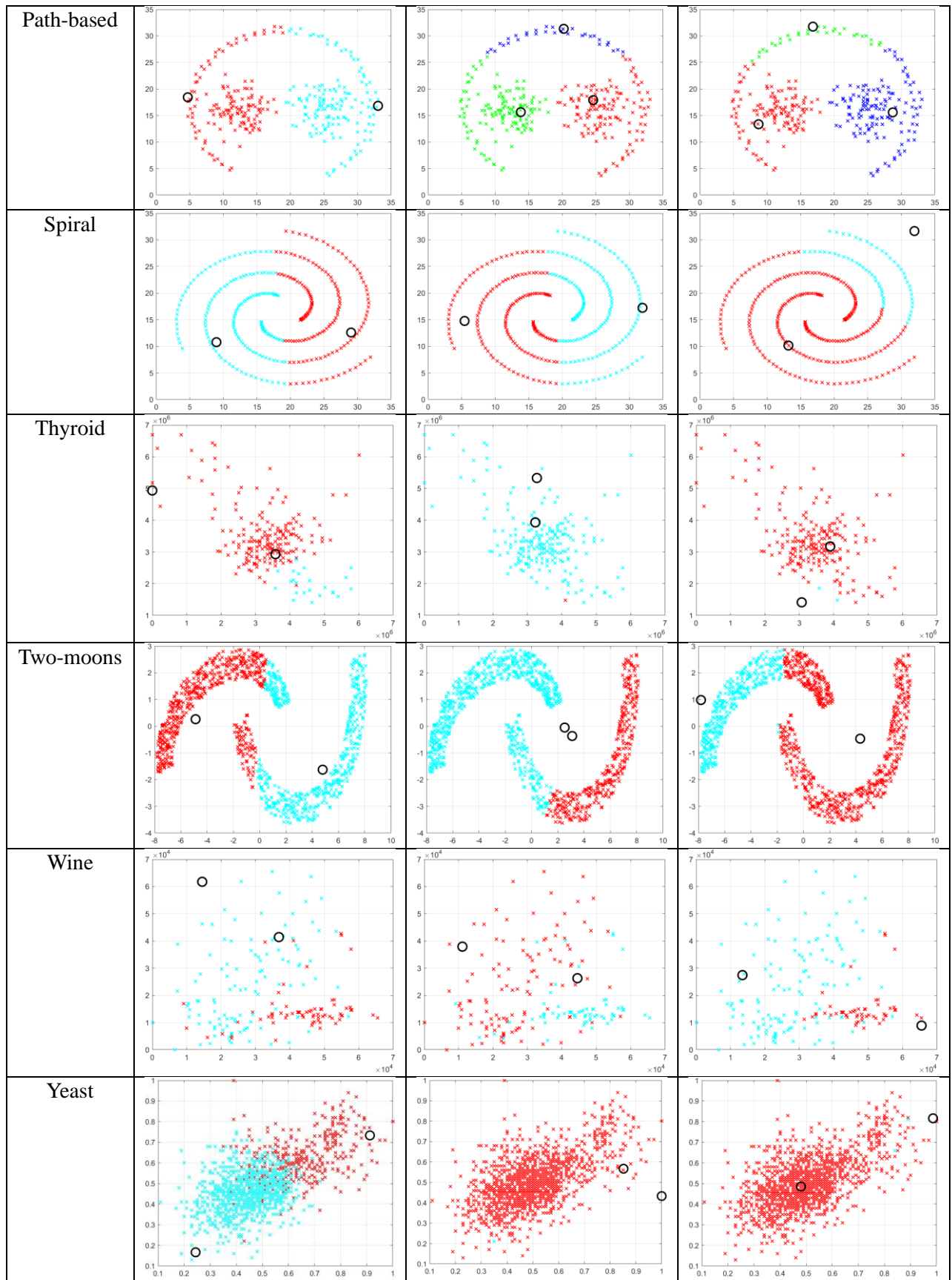

Supplement: Supplementary file 1 — Supplementary Material 1 [file 41598_2025_8473_MOESM1_ESM.pdf]
