# Supplementary material for: Benchmarking validity indices for evolutionary K-means clustering performance
Source: Sci Rep. 2025 Jul 1;15:21842. doi: 10.1038/s41598-025-08473-6 (PMC12218181; doi:10.1038/s41598-025-08473-6)
Supplement: Supplementary file 2 — Supplementary Material 2 [file 41598_2025_8473_MOESM2_ESM.docx]

**Appendix 1:** Clustering Results for twelve datasets based on CH index, Silhouette and SV index

| Dataset | CH Index | Silhouette Index | SV Index |
| --- | --- | --- | --- |
| Breast | 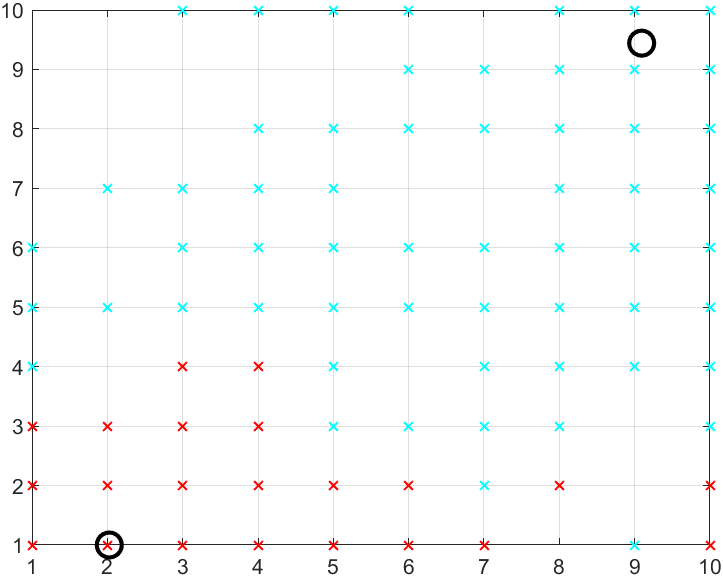 | 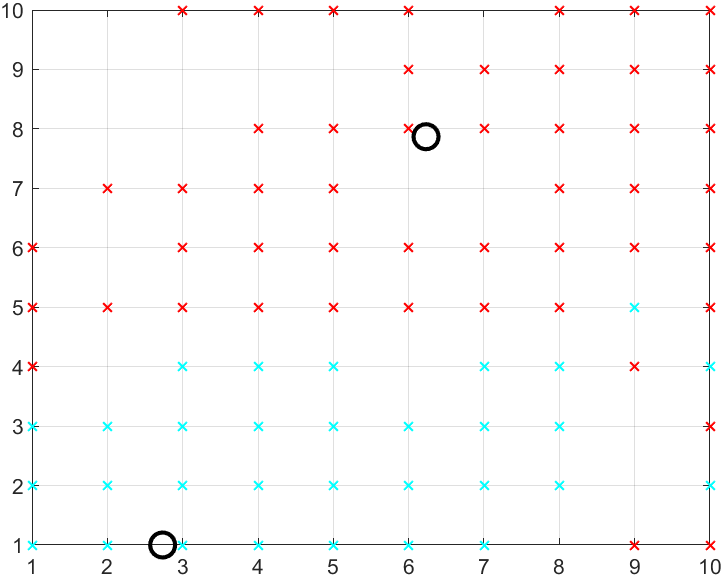 | 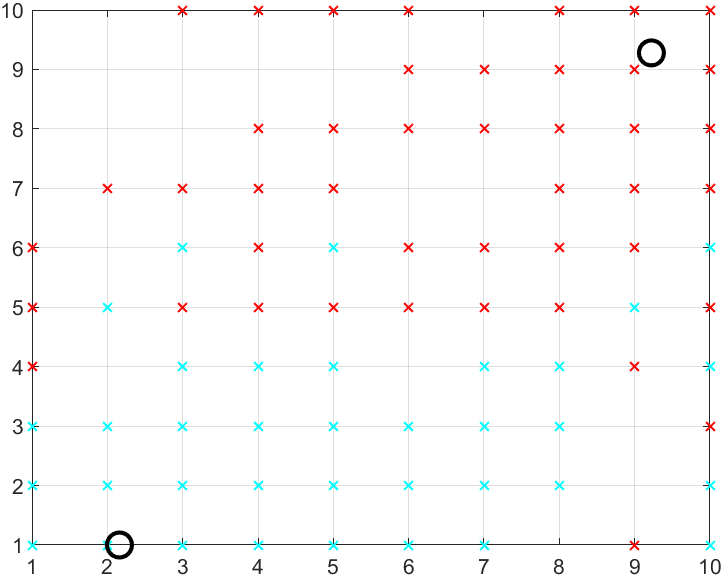 |
| Compound | 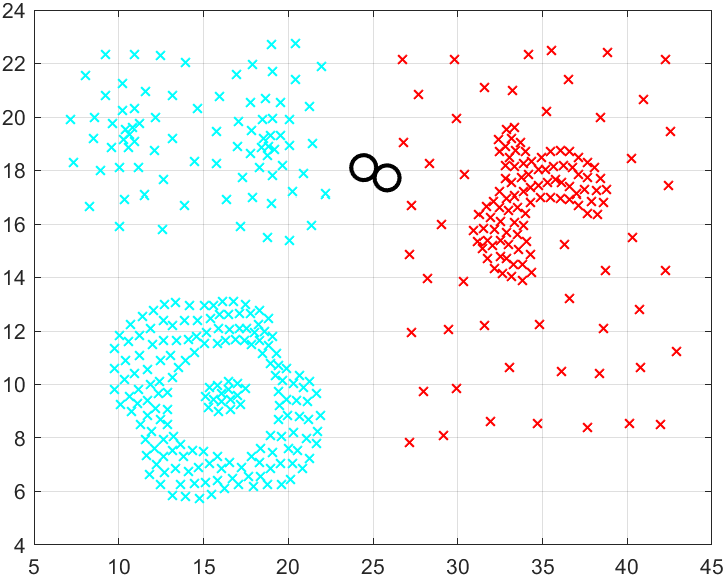 | 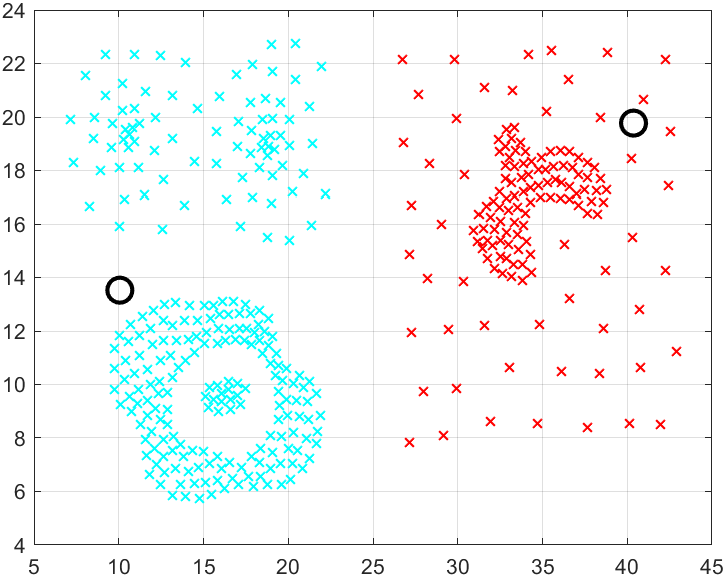 | 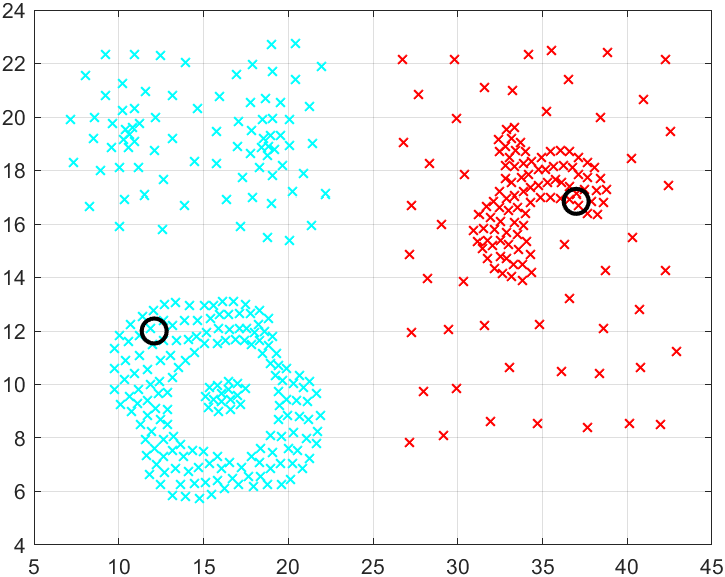 |
| Flame | 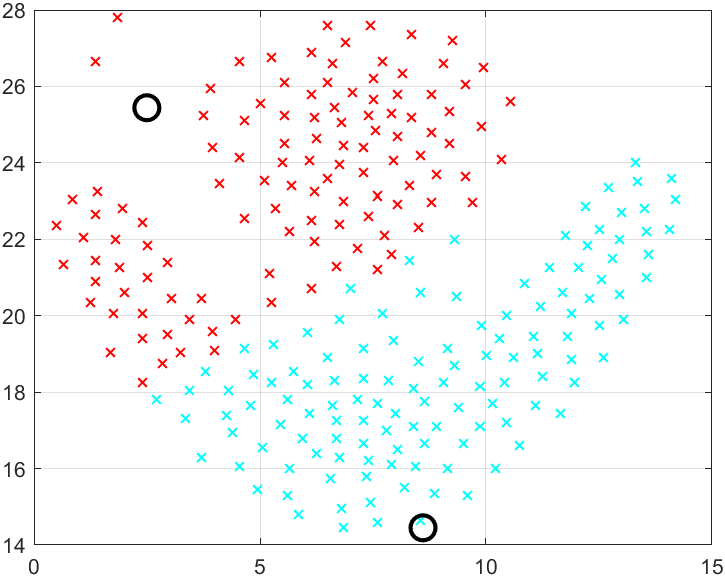 | 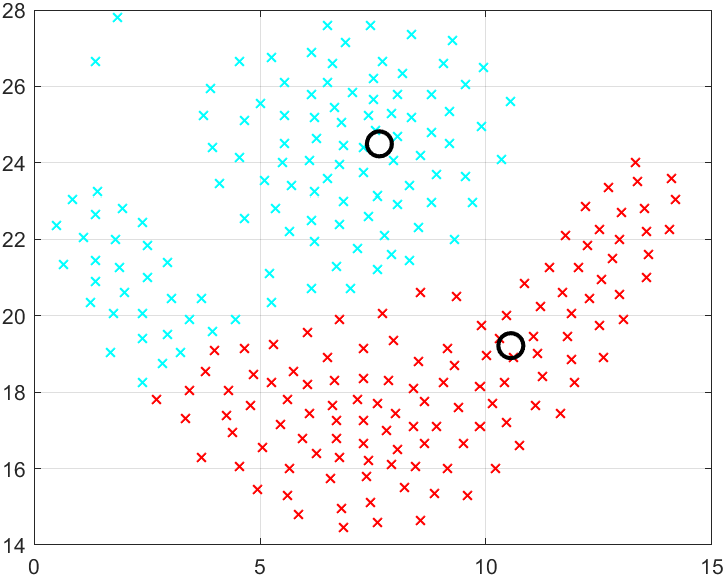 | 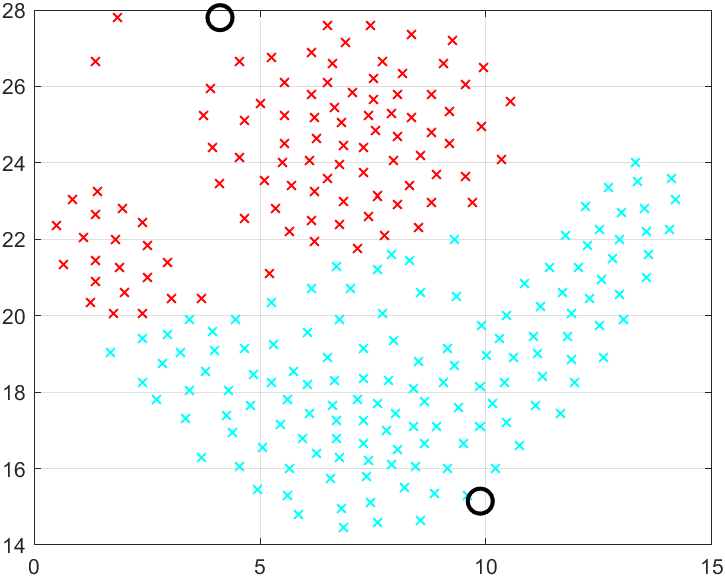 |
| Glass | 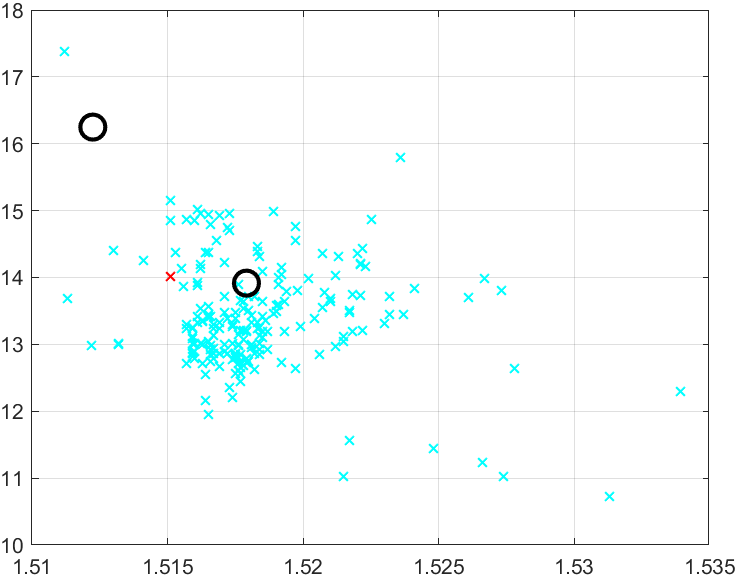 | 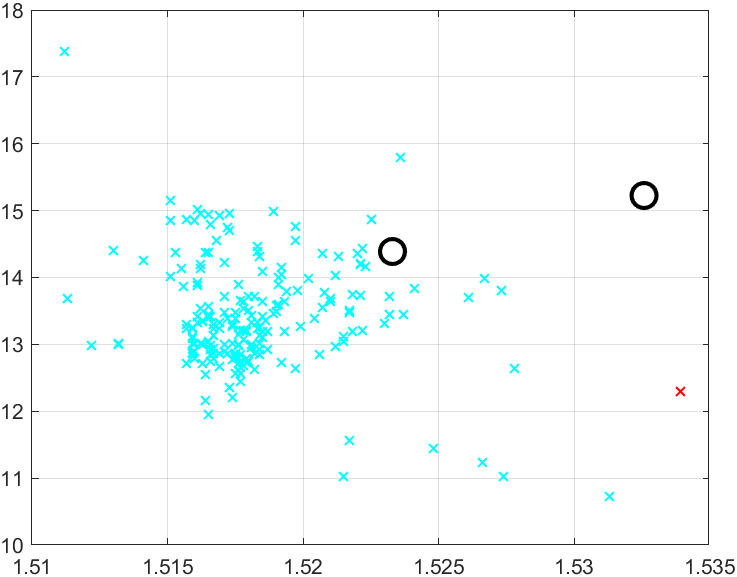 | 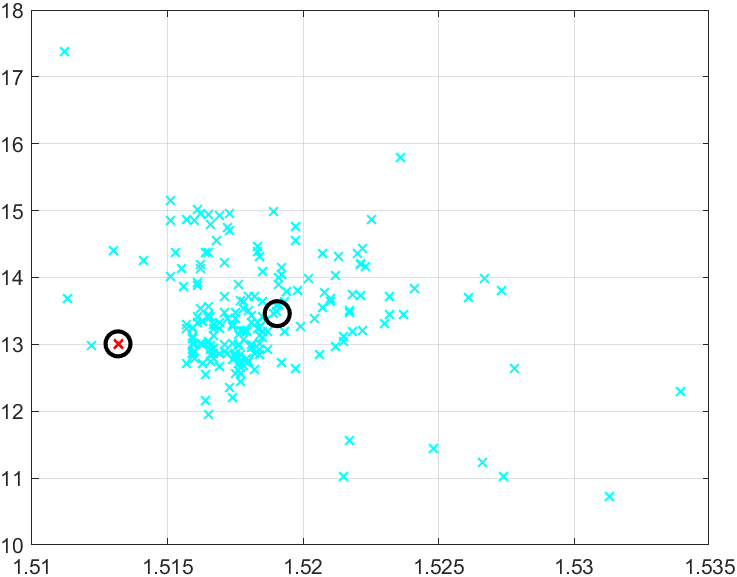 |
| Iris | 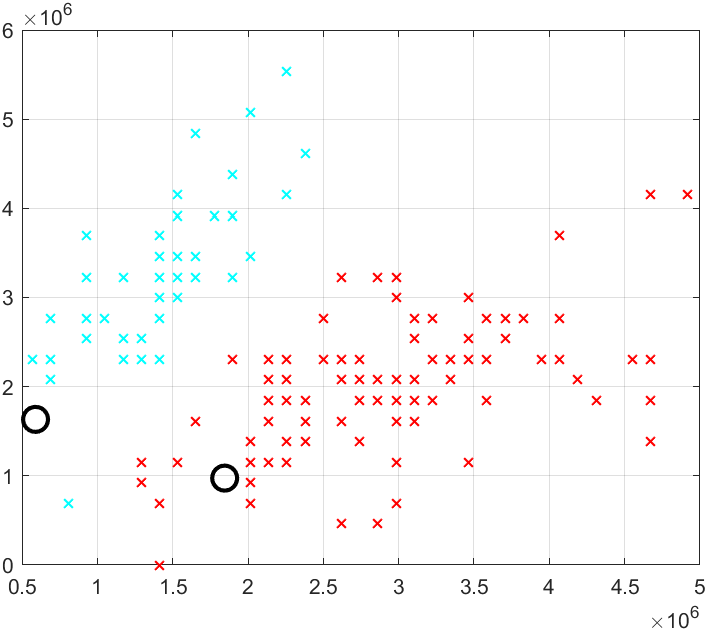 | 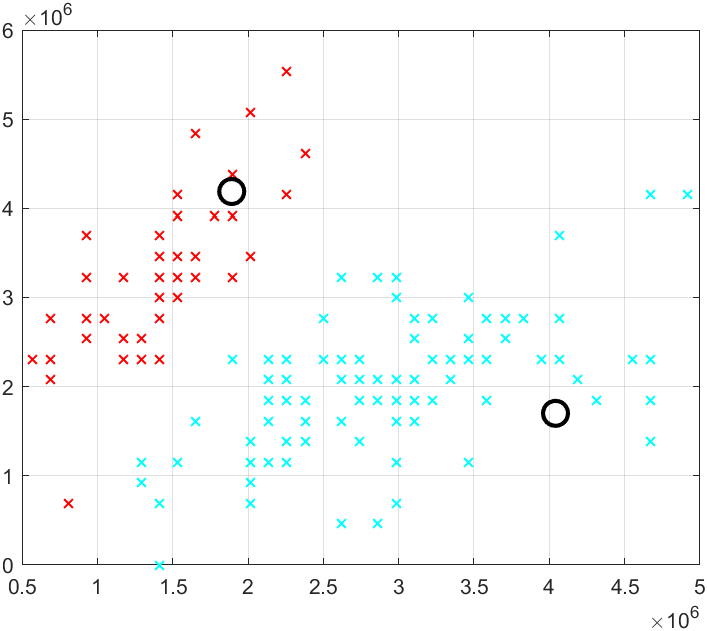 | 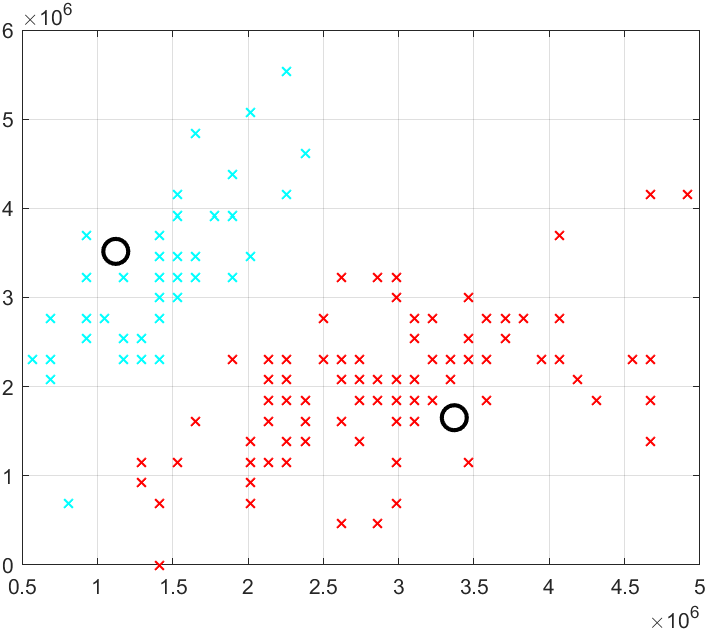 |
| Jain | 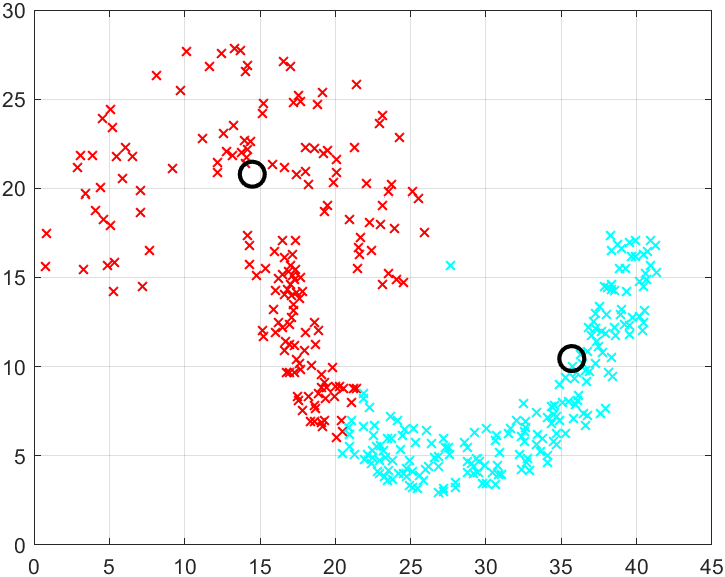 | 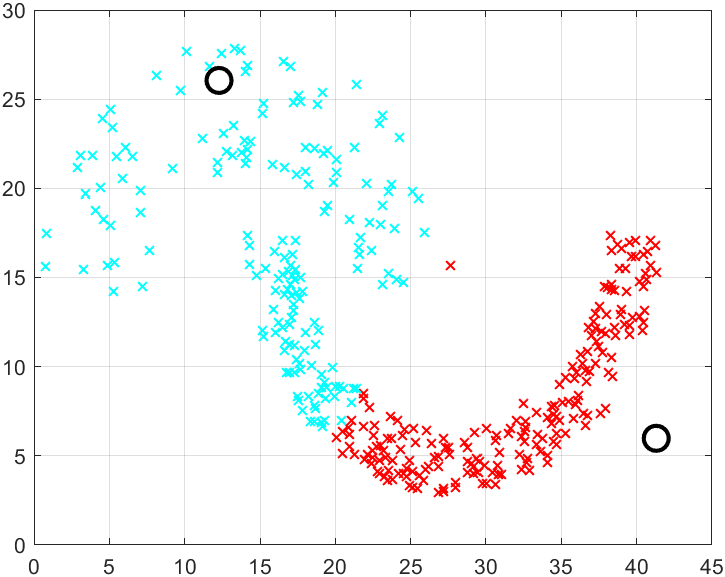 | 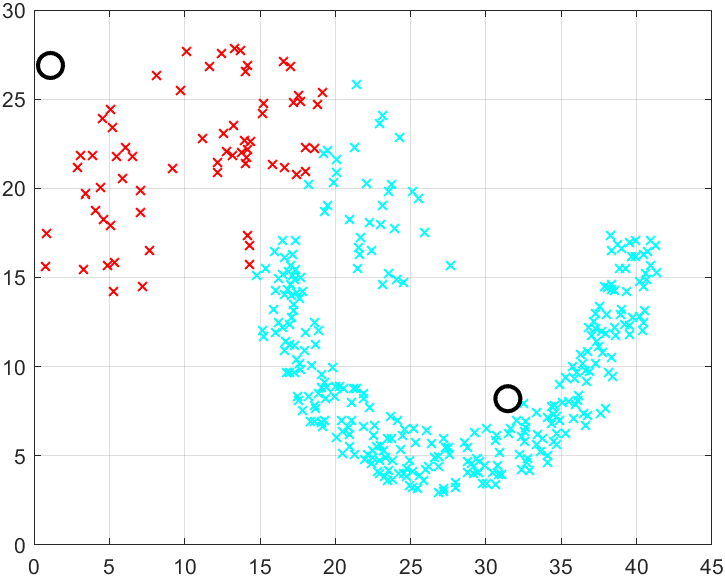 |
| Path-based | 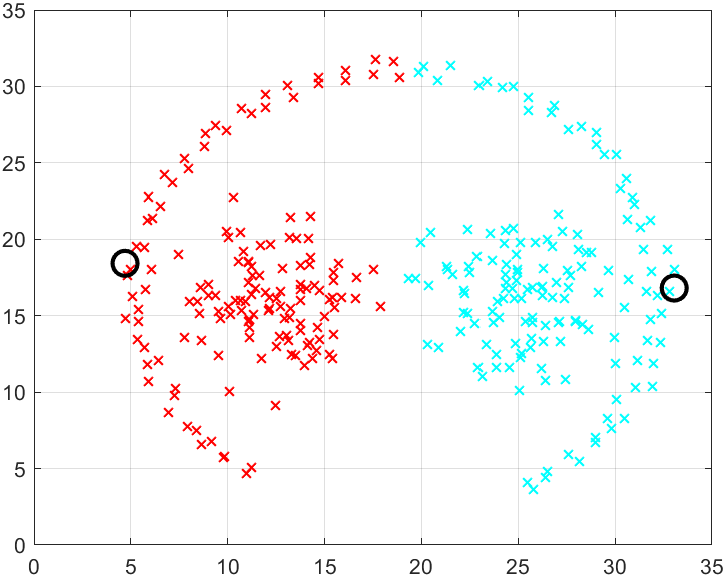 | 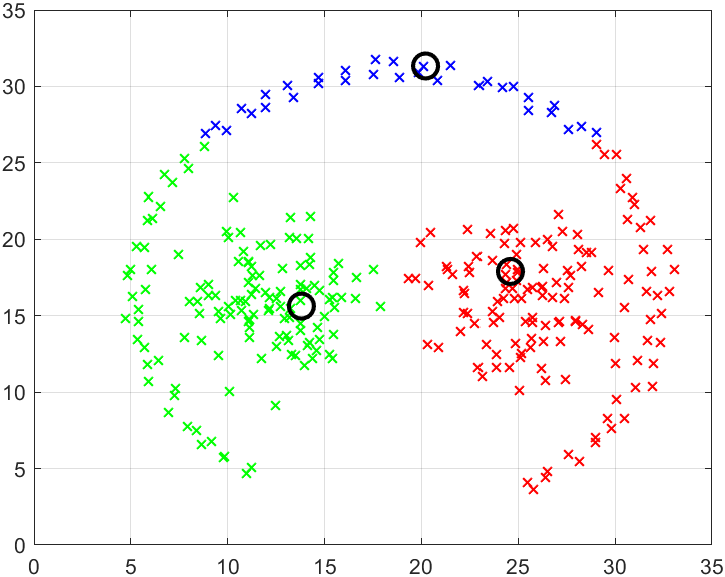 | 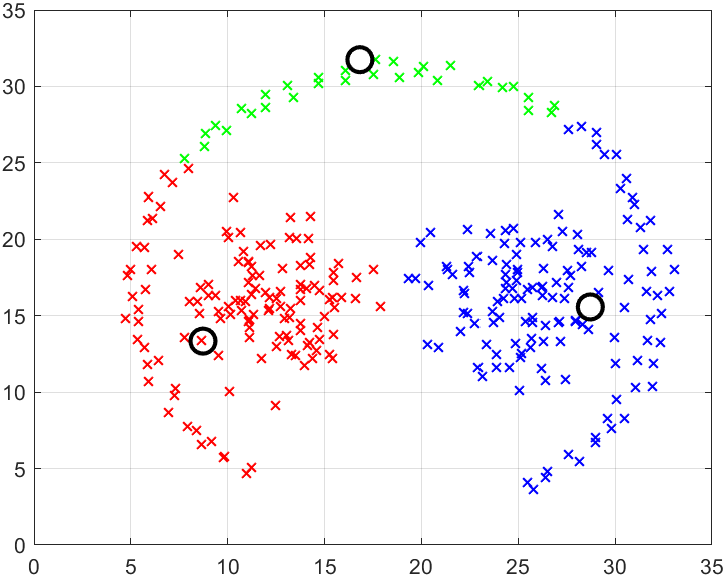 |
| Spiral | 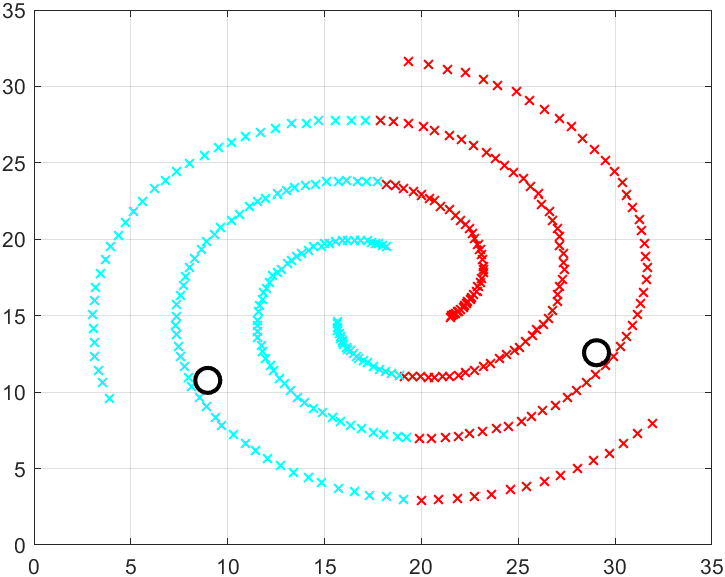 | 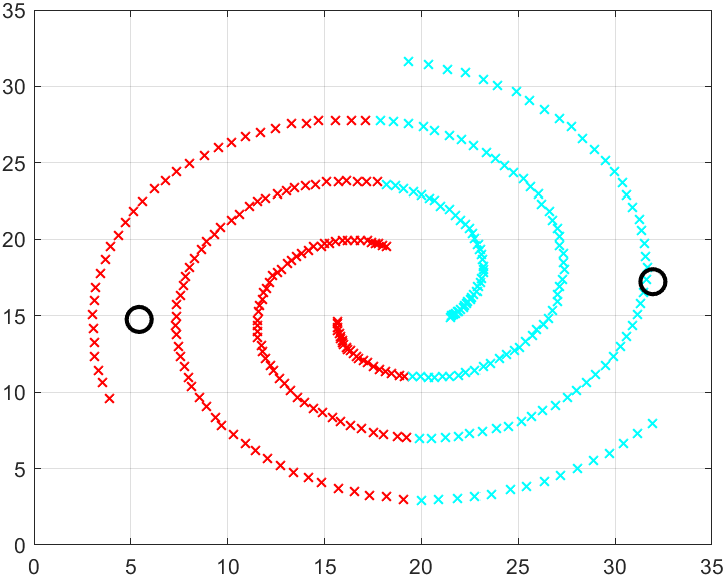 | 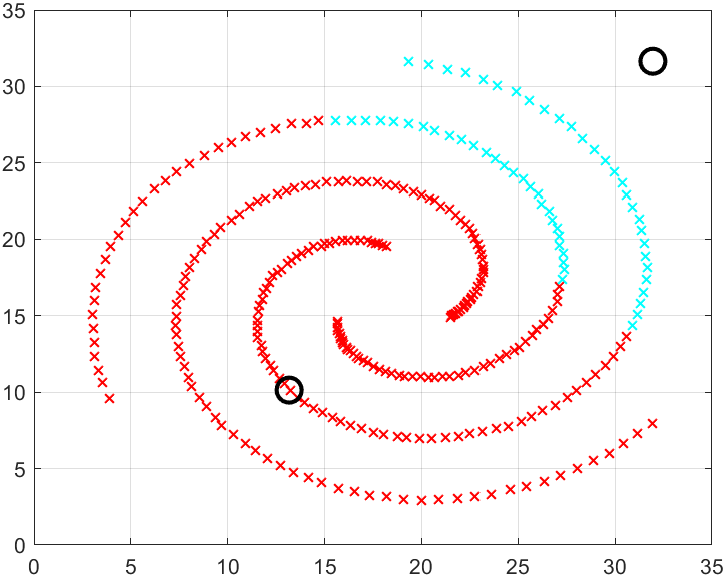 |
| Thyroid | 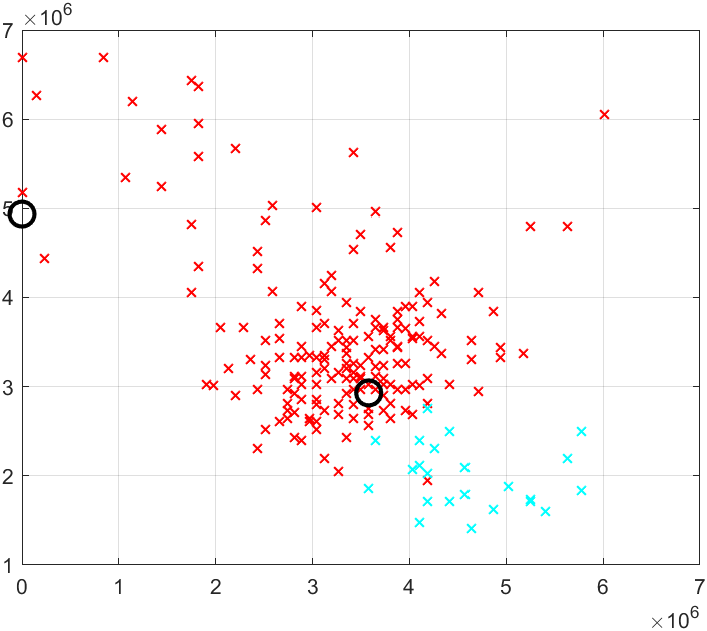 | 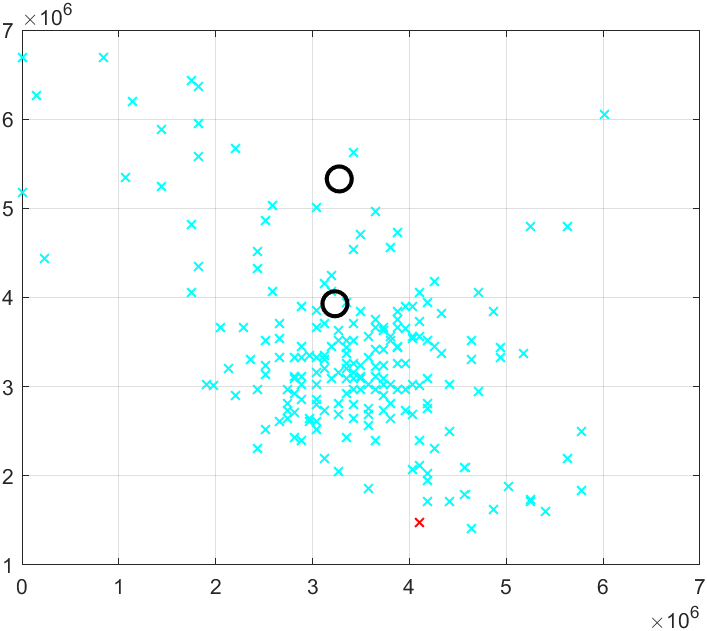 | 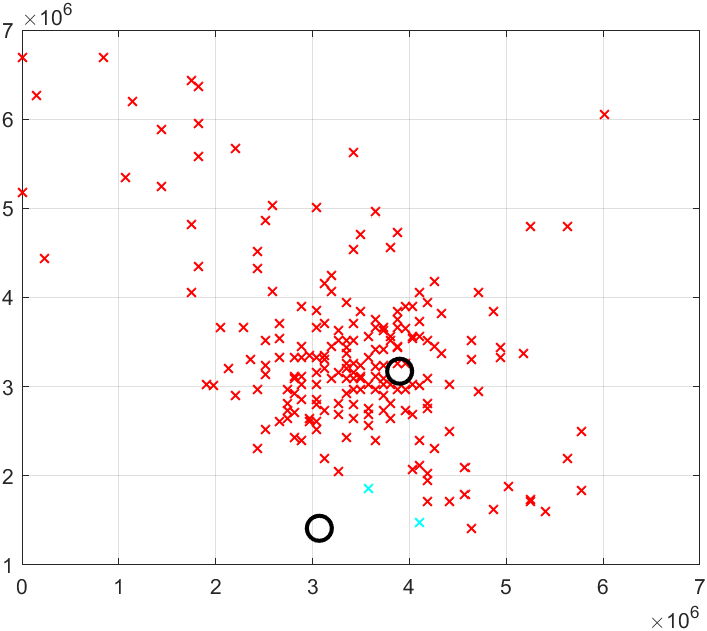 |
| Two-moons | 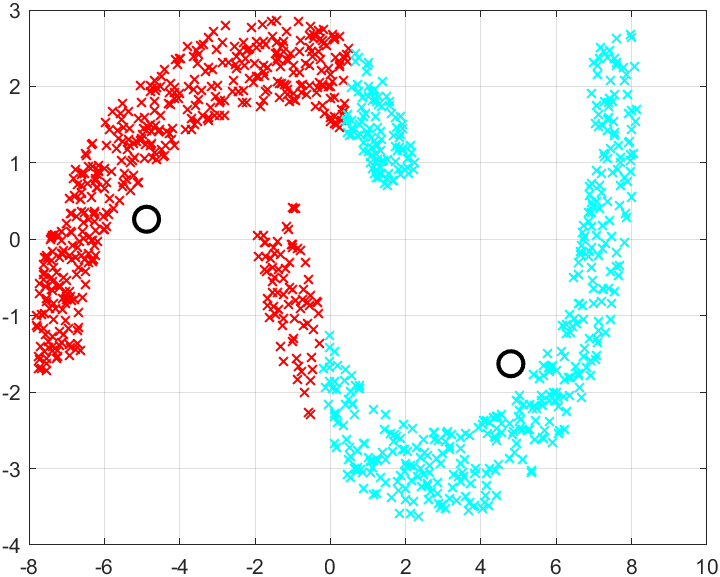 | 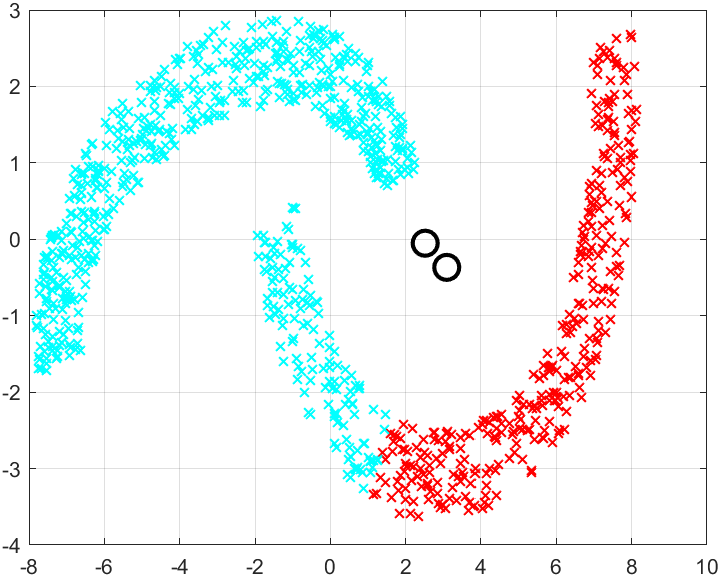 | 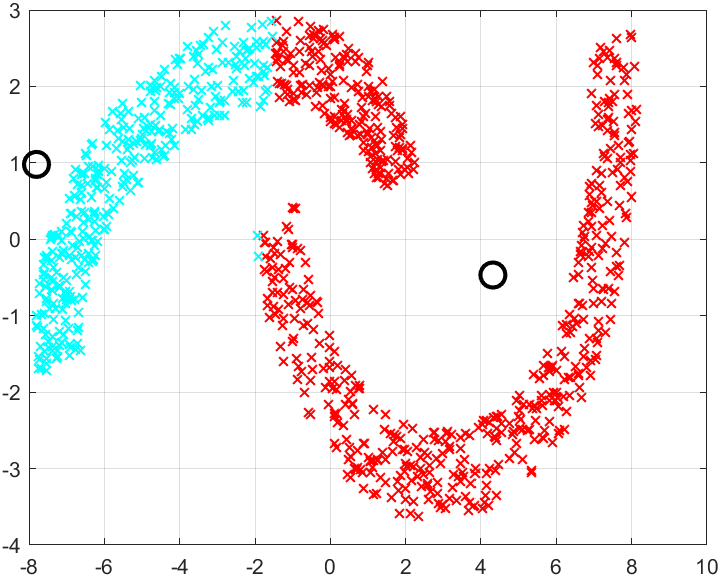 |
| Wine | 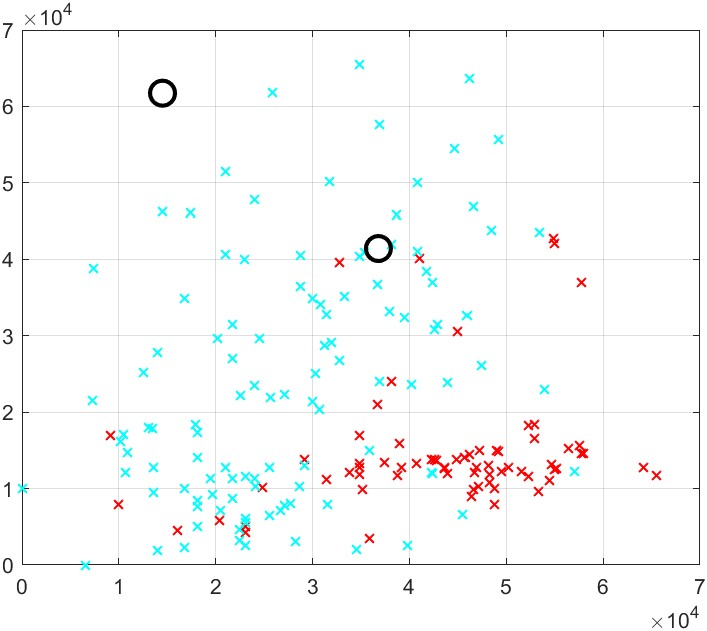 | 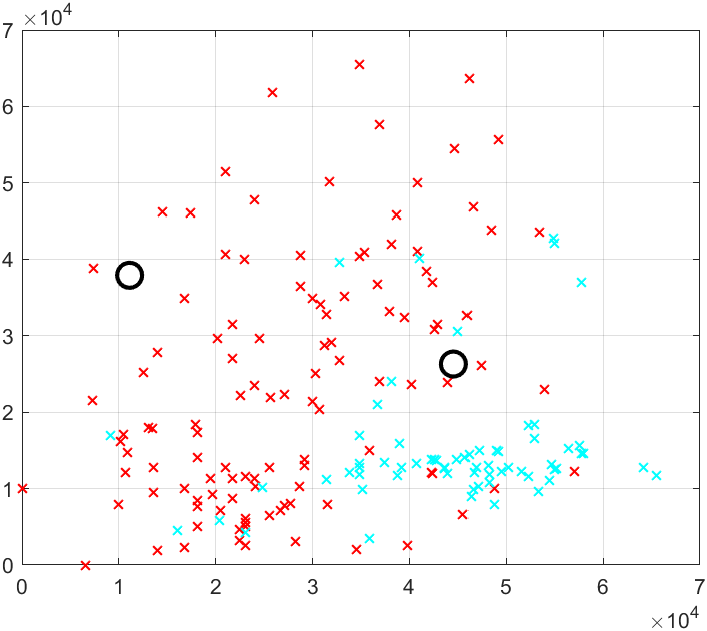 | 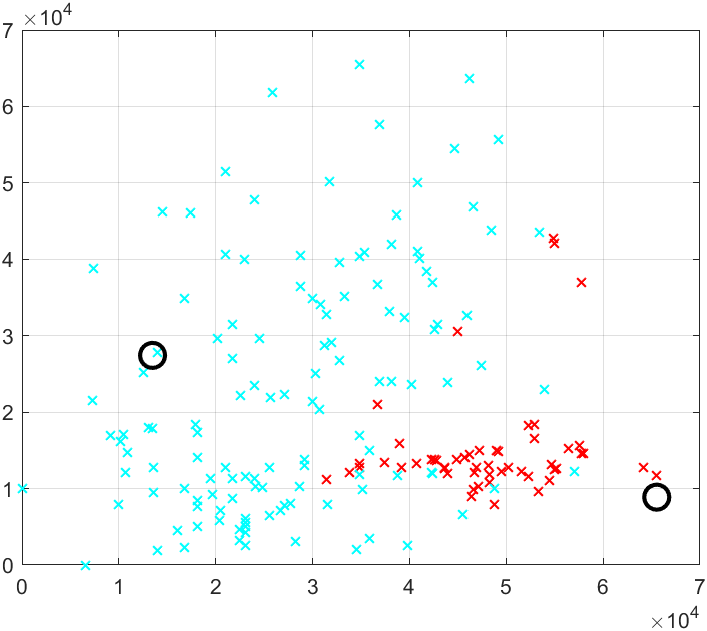 |
| Yeast | 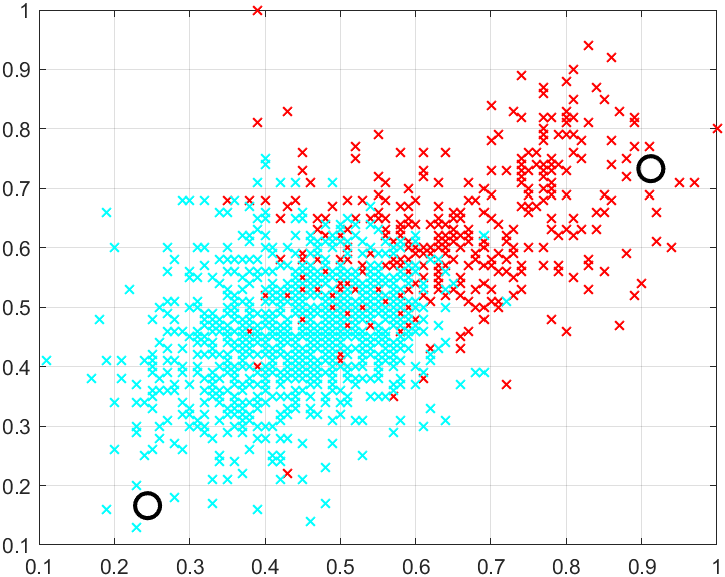 | 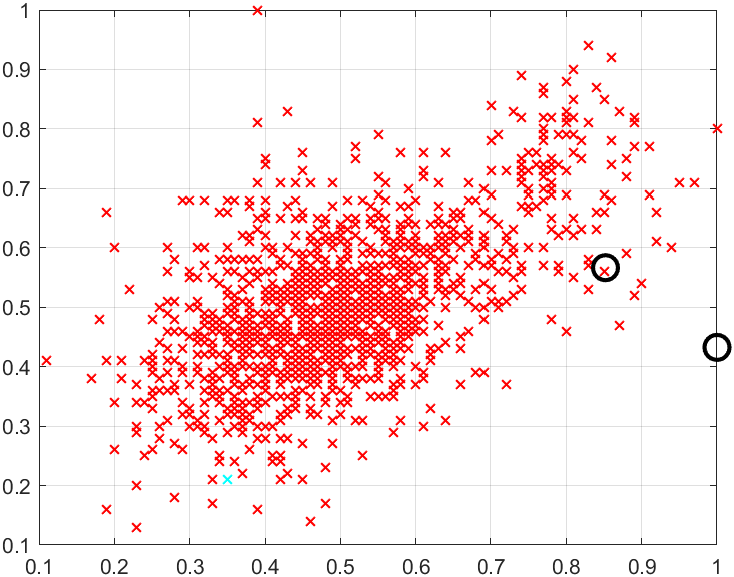 | 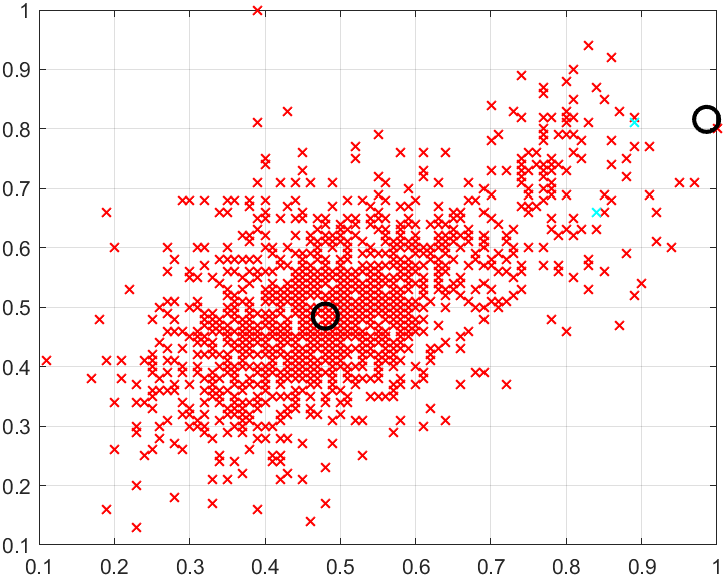 |
